# AI-Guided Histopathological and Molecular Phenotyping of Salivary Glands in Sjögren’s Disease: Subtyping, Mechanistic Insights, and Translational Implications—A Systematic Review and Structured Functional Synthesis

**DOI:** 10.3390/medsci14060677

**Published:** 2026-10-15

**Authors:** Carlos M. Ardila, Eliana Pineda-Vélez, Anny M. Vivares-Builes

**Affiliations:** 1 Department of Periodontics, Saveetha Dental College, and Hospitals, Saveetha Institute of Medical and Technical Sciences, Saveetha University, Chennai 600077, India; 2 Biomedical Stomatology Research Group, Basic Sciences Department, Faculty of Dentistry, Universidad de Antioquia U de A, Medellín 050010, Colombia; eliana.pineda@uam.edu.co (E.P.-V.); anny.vivares@uam.edu.co (A.M.V.-B.); 3 Faculty of Dentistry, Institución Universitaria Visión de las Américas, Medellín 050040, Colombia

**Keywords:** salivary gland pathology, Sjögren’s disease, artificial intelligence, machine learning, digital pathology, molecular phenotyping

## Abstract

Background/Objectives: Salivary-gland biopsy is central to Sjögren’s disease (SjD) assessment, but conventional measures incompletely capture glandular heterogeneity. This systematic review evaluated artificial intelligence (AI)/machine learning (ML) applications to SjD salivary-gland histopathology and molecular profiling, including biological and translational validation. Methods: PubMed, Scopus, and Embase were searched. Eligible studies used salivary-gland tissue as the primary analytical substrate or provided substantive glandular validation of systemic AI/ML findings. Evidence was synthesized functionally because of methodological heterogeneity. Results: Twenty-five studies were included. AI/ML identified histopathological and molecular phenotypes spanning immune/interferon activation, tissue architecture and immune-cell organization, epithelial stress and metabolic/cell-death remodeling, and translational response. Only a limited subset progressed beyond computational or tissue-level confirmation to functional perturbation, pharmacological testing, in vivo validation, or human treatment-response assessment. Recurrent public-dataset reuse, small cohorts, heterogeneous pipelines, and limited independent validation constrained reproducibility. Conclusions: AI/ML supports multidimensional SjD glandular phenotyping and shows emerging translational potential, but demonstrated clinical or therapeutic utility remains unestablished. Prospective multicenter validation is required before AI-derived glandular phenotypes can inform routine patient stratification or therapeutic decision-making.

## 1. Introduction

Sjögren’s disease (SjD) is a chronic systemic autoimmune disorder with a marked tropism for the exocrine glands, particularly the salivary and lacrimal glands [1]. Within head and neck pathology, the salivary gland is not merely an organ affected by a systemic disease but a primary site in which the characteristic tissue lesion can be directly assessed. Focal lymphocytic sialadenitis with a focus score of at least 1 focus per 4 mm^2^ of glandular tissue remains one of the highest-weighted components of the 2016 ACR/EULAR classification criteria [2]. Beyond this diagnostic role, labial salivary gland biopsy provides access to the epithelial, stromal, and immune compartments in which SjD evolves. These tissues may show acinar and ductal epithelial injury, organized T- and B-cell infiltrates, lymphoepithelial lesions, ectopic germinal-center-like structures, and variable fibrosis, reflecting a dynamic interaction between glandular epithelium and local immunity [3,4]. Accordingly, salivary gland pathology has increasing relevance not only for classification, but also for biological stratification and therapeutic-response assessment.

Conventional histopathological evaluation, however, captures only part of this complexity. Although standardized acquisition and reporting have improved the reproducibility of focus score assessment, substantial variability remains in tissue sampling, interpretation, and characterization of additional pathological features [3,5]. Importantly, incorporation of multiple histopathological features beyond the focus score can improve the information obtained from labial gland biopsy [6]. At the same time, molecular pathology has revealed that morphologically similar glands may contain markedly different epithelial and immune states. Single-cell transcriptomic analyses have identified heterogeneity among epithelial, endothelial, stromal, and immune populations and have implicated altered epithelial regeneration, lymphocyte trafficking, and stromal–immune interactions in glandular dysfunction [7]. These observations support a broader view of SjD pathology in which conventional morphology, immunophenotype, spatial organization, and tissue-level molecular programs are complementary layers of the same disease process. The resulting high-dimensional data also create an analytical challenge that is difficult to address with conventional univariate approaches alone.

Artificial intelligence (AI) and machine-learning (ML) methods offer a means of interrogating this multidimensional tissue landscape. In digital pathology, deep-learning models can analyze whole-slide images and, when coupled with explainability methods, identify histological patterns that extend beyond automated focus-score classification [8]. In molecular pathology, unsupervised learning has been used to define interferon-dominant glandular phenotypes [9] and to uncover clinically distinct subgroups from labial salivary gland DNA-methylation profiles [10]. Machine-learning approaches applied directly to glandular metabolomic data have likewise identified immunometabolic signatures associated with lymphocytic sialadenitis [11], while spatially resolved and multiplex tissue analyses are beginning to connect computationally derived patterns with cellular interactions and epithelial injury [12]. More recently, AI-guided feature prioritization has been linked to experimental mechanistic validation and tissue-level intervention in glandular models, illustrating how computationally derived phenotypes may acquire translational relevance [13,14]. Collectively, these applications suggest that AI can function not only as a diagnostic aid, but also as a discovery framework for pathology-based subtyping, mechanistic interpretation, and identification of potentially actionable tissue phenotypes.

Despite this expansion, the evidence remains fragmented across digital histopathology, transcriptomics, epigenomics, metabolomics, spatial analysis, and experimental models. A recent review summarized machine- and deep-learning applications for Sjögren’s syndrome diagnosis across histopathology, imaging, omics, and other data sources [15]. That diagnostic perspective is valuable, but it does not specifically address AI as a tool for understanding salivary gland pathology itself or for linking glandular phenotypes to disease mechanisms and their translational implications. This distinction is important because a large proportion of AI studies in SjD are based primarily on blood, peripheral blood mononuclear cells, serum, or clinical variables and therefore do not directly interrogate the diseased salivary gland. Conversely, tissue-centered studies are beginning to show that computational analysis can reveal biologically meaningful glandular subtypes, previously unrecognized histopathological patterns, mechanistically informative molecular states, and tissue responses to intervention. A synthesis focused specifically on this glandular pathological continuum is therefore needed to clarify what AI has contributed beyond diagnosis, how robustly these tissue phenotypes have been validated, and how far they have progressed toward clinically meaningful translation.

Accordingly, this systematic review aimed to evaluate how AI and ML approaches have been applied to the molecular and histopathological phenotyping of salivary glands in SjD, with particular emphasis on pathology-based subtyping, mechanistic interpretation, and the translational implications of AI-derived tissue phenotypes. A structured functional synthesis was undertaken to map the evidence across the continuum from glandular phenotype discovery and pathological subtyping to mechanistic validation and emerging therapeutic translation, while explicitly accounting for differences in tissue provenance, analytical function, validation depth, and reuse of public datasets or overlapping cohorts.

## 2. Materials and Methods

### 2.1. Protocol and Reporting Standards

This systematic review and structured functional synthesis was conducted in accordance with the Preferred Reporting Items for Systematic Reviews and Meta-Analyses (PRISMA) 2020 statement [16], with reporting of the literature search informed by PRISMA-S [17]. The review protocol was prospectively registered in PROSPERO (CRD420261490289). The review question, eligibility criteria, information sources, search strategy, evidence domains, and synthesis framework were prespecified before formal data extraction. Because the included literature spans digital histopathology, tissue-based omics, observational human studies, clinical prediction models, randomized treatment-response pathology, and preclinical experimental studies, the review was designed a priori around a structured functional synthesis rather than a single pooled effect estimate.

### 2.2. Eligibility Criteria

Peer-reviewed full-text original studies were eligible when they investigated SjD and AI or ML methods and made a substantive contribution to the analysis of salivary-gland pathology. Eligible outputs included molecular or histopathological phenotyping, pathology-based subtyping, feature or target prioritization, mechanistic interpretation, clinically relevant stratification, or tissue-linked treatment-response assessment. Human studies, secondary analyses of public salivary-gland datasets, digital pathology studies, translational studies, and integrated animal or cellular validation studies were eligible. No restrictions were imposed by publication year, language, country, clinical setting, or laboratory platform.

The salivary gland had to constitute a primary analytical or experimental substrate rather than serving only as a secondary validation dataset. Two routes to eligibility were prespecified. Under Path A, AI/ML was applied directly to salivary-gland histology, whole-slide images, transcriptomics, methylation or other epigenomic profiles, metabolomics/lipidomics, single-cell or spatial data, or other gland-derived molecular information. Under Path B, AI/ML discovery could originate in a systemic compartment only when the same study subsequently provided substantial direct experimental validation of the AI-derived finding in salivary-gland tissue or glandular cells. For Path B, the minimum acceptable validation required direct glandular evidence demonstrating that the AI-prioritized feature, pathway, or phenotype was biologically anchored to salivary-gland tissue or glandular cells and supported the same inference generated in the systemic discovery analysis. Eligibility was not determined by the assay type alone. Isolated confirmation that a candidate gene or protein was detectable or differentially expressed was insufficient. Immunohistochemical validation was considered adequate only when performed in independent glandular tissue and when it provided pathological localization or a tissue-level association relevant to the AI-derived finding. Similarly, qRT-PCR confirmation in a small glandular sample alone was not sufficient unless it formed part of a broader glandular validation showing biological or pathological relevance. For in vivo disease-model studies, this broader glandular validation could consist of direct measurement of the AI-prioritized feature in pathologically affected salivary-gland tissue together with histopathological evidence of glandular disease within the same experimental model. Such evidence was considered pathological anchoring rather than functional or causal validation. Acceptable Path B evidence therefore included independent glandular histopathological or immunohistochemical confirmation with pathological anchoring, glandular single-cell or spatial localization, in vivo disease-model confirmation meeting the pathological-anchoring criterion described above, or direct cellular or experimental perturbation supporting the same biological inference. Mere external receiver operating characteristic (ROC) testing, secondary computational replication, or expression confirmation in a public salivary-gland dataset was insufficient. Borderline cases were independently assessed by two reviewers at full text, and inclusion required agreement that the glandular validation materially supported the AI-derived biological inference; unresolved disagreements were adjudicated by a third reviewer. The Path A/Path B eligibility framework and the minimum requirement for substantive direct glandular validation under Path B were prespecified in the registered PROSPERO protocol.

Eligible computational methods included supervised and unsupervised learning, deep learning, neural networks, random forests, support vector machines, penalized learning/feature selection (including LASSO when used as part of a learning pipeline), gradient boosting/XGBoost, variational autoencoders, explainable AI methods, and hierarchical or consensus clustering when these methods generated biologically or pathologically meaningful phenotypes. Conventional bioinformatics without a substantive learning component, pathway enrichment alone, network pharmacology alone, descriptive microscopy, and manual use of the term “pattern recognition” without computational learning were excluded. Studies using AI only for diagnosis, focus-score automation, or case detection were excluded unless the analysis also generated an eligible molecular or histopathological phenotype, mechanistic insight, or pathology-based stratification output. Reviews, editorials, commentaries, protocols, registry-only records, conference abstracts or proceedings without a full report, preprints, and duplicate reports without a distinct eligible analysis were excluded.

To avoid treating all computational approaches as equivalent forms of AI/ML, eligibility required at least one data-driven learning component that performed algorithmic feature selection, classification or prediction, latent-pattern or subgroup discovery, or representation learning from empirical data. Conventional inferential statistics, differential-expression analysis, pathway enrichment, weighted gene co-expression network analysis, protein–protein interaction analysis, network pharmacology, or molecular docking alone were not considered sufficient to qualify a study as AI/ML. For descriptive reporting, eligible computational methods were grouped into five non-mutually exclusive classes: (1) conventional feature-selection/statistical-learning methods, including penalized regression such as LASSO or elastic-net approaches; (2) classical machine-learning methods, including random forests, support-vector machines, naïve Bayes classifiers, decision-tree methods, and gradient-boosting algorithms; (3) unsupervised-learning methods, including hierarchical or consensus clustering used for data-driven phenotype or subgroup discovery; (4) deep-learning methods, including convolutional neural networks, multiple-instance learning, vision transformers, and variational autoencoders; and (5) explainable-AI methods, including SHAP/Shapley attribution and attention- or pattern-level attribution. Hybrid studies could contribute to more than one class. Ancillary bioinformatic analyses did not determine AI/ML eligibility unless an eligible learning component was also present.

### 2.3. Information Sources and Search Strategy

PubMed/MEDLINE, Scopus, and Embase were searched from database inception through 15 August 2026. The strategies combined controlled vocabulary, where available, with free-text terms for SjD, AI/ML, and salivary-gland, histopathological, molecular, mechanistic, and translational concepts. No date, language, human-only, or publication-type filters were applied at the search stage. Google Scholar was used as a supplementary sensitivity source. The screening of the first 200 results ranked by relevance was prespecified in the registered PROSPERO protocol as a pragmatic supplementary-search threshold because Google Scholar retrieves a very large and progressively less relevant result set and does not provide the same reproducible structured searching and export functionality as bibliographic databases. This supplementary search was intended to identify potentially relevant records not captured by the three primary databases rather than to serve as an exhaustive database search. Backward reference-list checking and forward citation searching were performed for eligible and potentially eligible reports. Registry records encountered during the search were checked for linked publications but were not included as evidence in the absence of a full peer-reviewed report. The complete database-specific strategies and supplementary-search procedures are provided in Supplementary Table S1.

### 2.4. Study Selection

Records from the three bibliographic databases were merged and deduplicated in Microsoft Excel (Microsoft Corporation, Redmond, WA, USA) using DOI, PMID, normalized title, and, where applicable, trial-registration identifiers. Two reviewers independently screened all titles and abstracts against the prespecified eligibility criteria. Titles and abstracts were evaluated against the prespecified criteria using a deliberately sensitive screening rule: records were retained for full-text assessment whenever the abstract did not allow tissue provenance, the substantive role of AI/ML, or translational relevance to be determined with confidence. The same two reviewers independently assessed the retrieved full texts against the complete Path A/Path B eligibility framework. Disagreements at either screening stage were resolved through discussion and, when consensus could not be reached, by adjudication by a third reviewer. Reports identified by backward or forward citation searching were assessed in parallel using the same independent full-text assessment procedure and retained as a separate “other methods” source for PRISMA reporting. Reasons for exclusion after full-text assessment were documented at the report level and are presented in Supplementary Table S2.

### 2.5. Data Extraction and Evidence Coding

A standardized extraction form was developed and piloted before formal extraction using Microsoft Excel (Microsoft Corporation, Redmond, WA, USA). Data from each included study were extracted independently by two reviewers using the standardized form. Extracted information was subsequently compared, and discrepancies were resolved through discussion; unresolved disagreements were adjudicated by a third reviewer. For each included study, the following items were recorded: bibliographic details; study design and setting; participant, animal, or cellular population; SjD definition or classification criteria; salivary-gland type and tissue source; sample size; source cohort or public-dataset accession; tissue preparation and histopathology, immunohistochemistry, immunofluorescence, or spatial methods; molecular/omics platform; AI/ML algorithm(s); intended analytical function; preprocessing and feature-selection procedures; training, internal-validation, and test procedures; validation setting; model performance when relevant; calibration when reported; interpretability/explainability; AI-derived histopathological or molecular phenotype; disease subtype; immune or epithelial correlate; pathway or candidate target; experimental perturbation; pharmacological or biologic intervention; glandular functional outcomes; treatment-response pathology; and author-reported limitations.

Validation setting was coded separately from biological or translational validation depth. Four analytical-validation categories were distinguished: (1) internal validation, defined as resampling, cross-validation, or train/validation/test partitioning within the same source cohort or dataset; (2) external public-dataset validation, defined as evaluation in a separately accessioned public dataset not used for model fitting; (3) independent clinical-cohort validation, defined as evaluation in separately recruited patients from a clinical cohort not used for model development, whether retrospective or prospective; and (4) prospective multicenter validation, defined as evaluation in prospectively recruited independent participants across multiple clinical centers under a prespecified validation framework. Public datasets with known or plausible participant overlap with the development data were classified as dataset reuse rather than independent external validation. These categories were reported separately and were not treated as equivalent forms of validation.

AI/ML outputs were coded according to their functional role rather than algorithm name alone. Thus, the same algorithm could contribute to different evidence domains depending on whether it was used for tissue classification, unsupervised subtyping, feature prioritization, mechanistic discovery, or treatment-response assessment. For mixed studies, the specific component that supported inclusion was identified explicitly so that diagnostic performance was not conflated with mechanistic or translational evidence.

### 2.6. Assessment of Dataset and Cohort Overlap

Because several studies reused public transcriptomic datasets, registries, or previously reported patient cohorts, dataset and cohort overlap was assessed explicitly. GEO accession numbers, named registries, recruitment centers, participant numbers, tissue sources, and overlapping time frames were extracted and cross-mapped in a dataset–study overlap matrix. Publications were retained when they provided analytically distinct eligible contributions, but overlapping participants were not summed as independent observations. Apparent replication based solely on reuse of the same dataset was distinguished from replication in biologically independent cohorts. Convergence of molecular pathways, targets, or phenotypes was therefore interpreted more strongly when supported by independent datasets, distinct tissue modalities, or experimental validation.

### 2.7. Functional Evidence Framework and Outcomes

The primary outcome was the identification and characterization of AI/ML-derived molecular or histopathological phenotypes of SjD-affected salivary glands and their pathological significance. Evidence was organized into five prespecified functional domains: (1) AI-guided histopathological and digital-pathology phenotyping; (2) molecular and multi-omic glandular phenotyping; (3) pathology-based subtyping and immune–epithelial states; (4) mechanistic insights derived from AI-prioritized features, pathways, genes, miRNAs, proteins, metabolites, or cell states; and (5) translational implications, including functional perturbation, pharmacological validation, treatment stratification, and pathology- or function-linked treatment response.

For each finding, validation depth was coded according to a six-level hierarchy used in the revised synthesis: (1) computational discovery, including internal resampling, cross-validation, or within-cohort validation when performed; (2) external public-dataset validation, defined as confirmation in a separately accessioned public dataset not used for model fitting and without known participant overlap; (3) independent clinical-cohort validation, defined as validation or biological confirmation in separately recruited human participants not used for model development, including independent salivary-gland tissue cohorts; (4) functional validation through direct perturbation or rescue in cellular or ex vivo systems; (5) in vivo validation through confirmation or intervention in an animal model; and (6) prospective multicenter clinical validation, defined as prospective evaluation of an AI/ML-derived phenotype, model, biomarker, or stratification finding in independently recruited participants across multiple clinical centers under a prespecified validation framework. Each finding was assigned the highest level directly supported by the study. Progression within this hierarchy reflected increasing biological and translational validation but was not interpreted as a numerical quality score, a formal certainty-of-evidence rating, or evidence that studies reaching a higher level necessarily had lower risk of bias.

To facilitate study-level interpretation of analytical independence and validation depth, findings were additionally assigned a non-scored descriptive validation-context indicator. Independent/experimental support (IE) denoted validation in a separately recruited human clinical cohort and/or direct functional, experimental, or interventional validation of the AI/ML-derived finding. Validation in an external public dataset alone did not qualify as IE. Direct tissue support (DT) denoted direct or orthogonal salivary-gland tissue confirmation without independent clinical-cohort or functional validation, including small confirmatory tissue series; and computational convergence (CO) denoted findings supported predominantly by secondary or public-dataset analyses without independent glandular biological confirmation. These categories were used only to make differences in validation context transparent and were not considered numerical measures of study quality or certainty of evidence.

Secondary outcomes included model discrimination, sensitivity, specificity, accuracy, F1 score, calibration, external validation, explainability, salivary flow, glandular functional markers, immune-cell composition, cytokines, oxidative-stress and apoptosis measures, and other tissue-level readouts when they were linked to an otherwise eligible pathological question.

### 2.8. Risk of Bias and Methodological Quality Assessment

Risk of bias and methodological quality were assessed using design-appropriate instruments rather than applying a single tool across the heterogeneous evidence base. The randomized clinical trial was evaluated using the Cochrane Risk of Bias 2 (RoB 2) tool, focusing on the intervention-effect result relevant to salivary-gland pathology [18]. Studies whose primary contribution was the development or evaluation of a participant-level prediction or classification model were assessed using the Prediction model Risk Of Bias ASsessment Tool + AI (PROBAST + AI), applying the model-development and/or model-evaluation components as appropriate [19]. Observational human tissue studies and secondary analyses of human salivary-gland datasets that did not primarily evaluate a clinical prediction model were assessed using the revised Joanna Briggs Institute (JBI) critical appraisal tool for analytical cross-sectional studies [20]. For analyses based on archived public datasets, items that were genuinely inapplicable were prespecified as not applicable rather than being judged negatively. Controlled animal intervention studies in which an in vivo therapeutic effect materially supported the eligible inference were assessed using the Systematic Review Centre for Laboratory animal Experimentation (SYRCLE) risk-of-bias tool [21]. For mixed translational studies, the instrument was applied to the study component that most directly supported the eligible inference, whereas ancillary animal, cellular, molecular, or tissue-validation experiments were characterized separately.

Because many included studies used AI/ML for exploratory feature selection, unsupervised phenotyping, molecular prioritization, or mechanistic discovery rather than for clinical prediction, no single validated AI-specific risk-of-bias instrument was applicable to the entire corpus. Accordingly, a parallel, non-scored AI methodological-safeguards matrix was applied descriptively to all studies. Prespecified items included data provenance and independence; sample size and class balance; transparency of preprocessing; separation of feature selection from model evaluation; potential information leakage; internal validation; independent or external validation; uncertainty estimation and calibration when applicable; interpretability; availability of data, code, or models; and biological or pathological validation. These domains were informed by PROBAST + AI and the Minimum Information about Clinical Artificial Intelligence Modeling (MI-CLAIM) checklist [19,22], but were not combined into an unvalidated composite quality score. All design-specific risk-of-bias and methodological-quality assessments using RoB 2, PROBAST + AI, JBI, and SYRCLE were performed independently by two reviewers. Disagreements were resolved through discussion and, when consensus could not be reached, by adjudication by a third reviewer. Item-level assessments and the rationale supporting the overall interpretation are provided in Supplementary Tables S3 and S4.

### 2.9. Reporting Bias and Confidence in the Evidence

Protocols, registry entries, Supplementary Files, and consistency between reported methods and results were examined when available to identify potential selective reporting, particularly when validation, calibration, histopathological, mechanistic, or therapeutic analyses were described but incompletely reported. Funnel plots and statistical tests for small-study effects were not planned because the included evidence did not provide a sufficiently homogeneous set of independent effect estimates for meaningful quantitative assessment of publication bias.

A formal Grading of Recommendations Assessment, Development and Evaluation (GRADE) assessment was not undertaken [23]. GRADE was developed primarily to assess certainty in bodies of evidence addressing defined clinical questions and outcomes, whereas the present review integrates heterogeneous exploratory molecular-pathology, histopathological, prediction-model, preclinical, and translational evidence rather than a single intervention-effect or diagnostic-effect question. Instead, confidence in each major synthesized finding was interpreted narratively according to study-level risk of bias and methodological quality; directness to salivary-gland pathology; independence of datasets or cohorts; consistency across studies and analytical modalities; and the depth of biological, histopathological, functional, or therapeutic validation.

### 2.10. Data Synthesis

A conventional quantitative meta-analysis was not performed because the included studies differed substantially in tissue modality, AI task, phenotype definition, molecular target, validation strategy, pathological outcome, and experimental design, and therefore did not provide sufficiently comparable independent effect estimates. The primary synthesis consisted of a structured functional synthesis, organized according to the prespecified functional evidence domains rather than by algorithm type. Reporting was informed, where applicable, by relevant principles of the Synthesis Without Meta-analysis (SWiM) guideline [24].

After findings had been organized within the five prespecified functional evidence domains, recurrent cross-cutting biological and translational themes were identified inductively by comparing the principal glandular processes represented across studies. These emergent themes comprised immune/interferon activation, tissue architecture and immune-cell organization, epithelial stress and cell-death/metabolic remodeling, and translational response. They were used as an integrative framework for the final cross-study synthesis and did not replace or modify the five prespecified functional evidence domains.

Within each domain, the synthesis considered the direction and biological meaning of the findings, degree of direct anchoring to salivary-gland pathology, independence of the underlying population or dataset, external or independent replication, interpretability, and depth of validation. Histopathological, molecular, multi-omic, mechanistic, and translational findings were therefore integrated according to their functional relationship rather than treated as equivalent outputs of AI/ML analyses.

Particular emphasis was placed on distinguishing independent convergence from apparent replication caused by reuse of the same public datasets or overlapping cohorts. Findings reproduced in independent populations, supported by orthogonal tissue modalities, or corroborated through direct experimental validation were given greater interpretive weight than findings reproduced solely through repeated analysis of the same datasets. Discordant, negative, or non-replicated findings were retained in the synthesis. The final structured functional synthesis was designed to map the evidence continuum from AI-derived glandular phenotype discovery and pathology-based subtyping through mechanistic interpretation to emerging translational implications, without implying that computational target prediction, database-based drug prioritization, or molecular docking alone constituted biological or therapeutic validation.

## 3. Results

### 3.1. Study Selection

**Figure 1:**
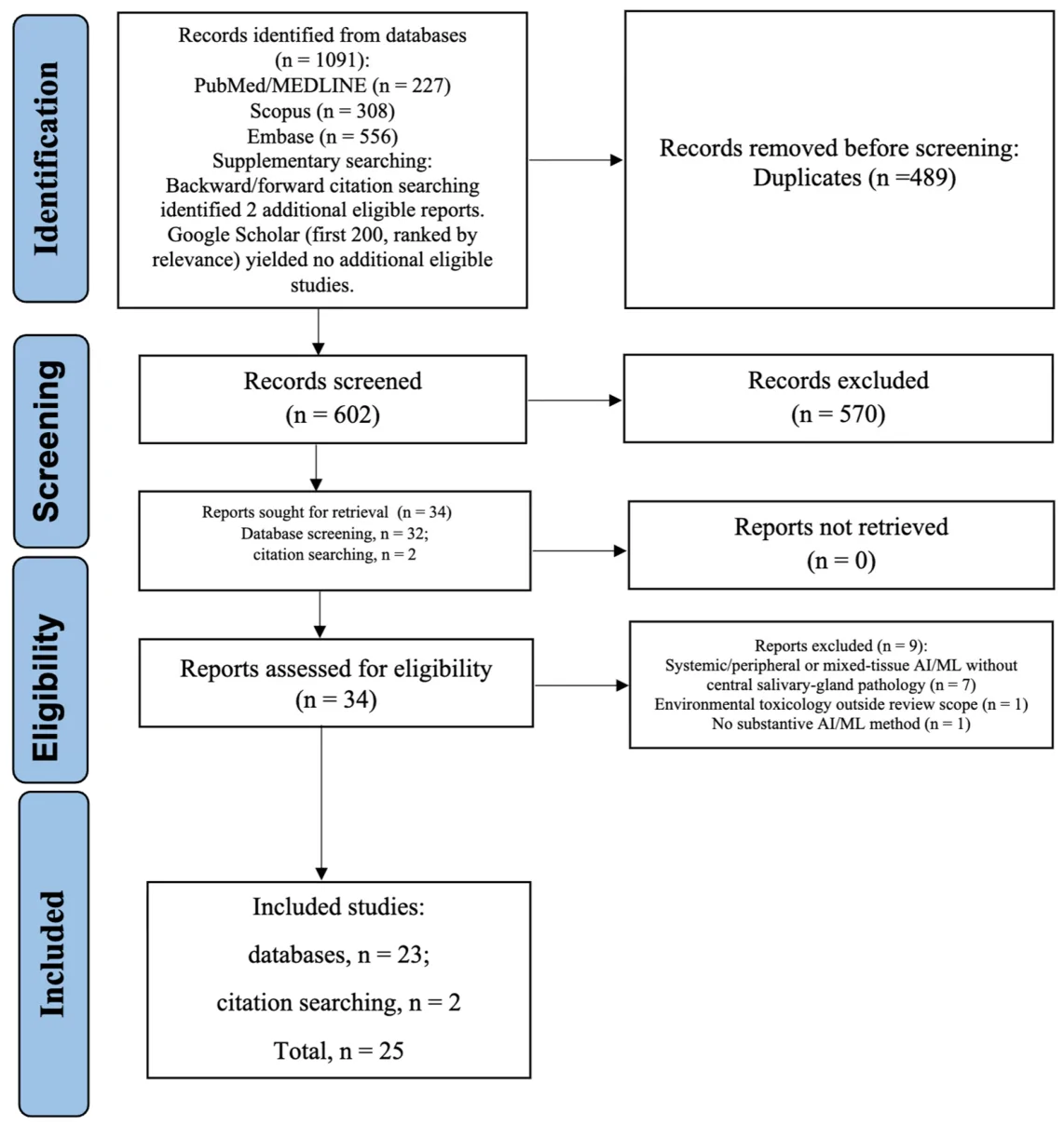
PRISMA 2020 flow diagram of the study-selection process.

The searches of PubMed, Scopus, and Embase identified 1091 records (227, 308, and 556, respectively). After removal of 489 duplicates, 602 unique records underwent title and abstract screening, of which 570 were excluded. Thirty-two reports were retrieved for full-text assessment. Backward and forward citation searching identified two additional potentially eligible reports, resulting in 34 full-text reports assessed for eligibility. Nine reports were excluded after full-text evaluation for prespecified reasons (Supplementary Table S2), leaving 25 studies [8,9,10,11,12,13,14,25,26,27,28,29,30,31,32,33,34,35,36,37,38,39,40,41,42] for inclusion in the systematic review and structured functional synthesis. In addition, the first 200 Google Scholar results, ranked by relevance, were screened and yielded no additional eligible studies. The study-selection process is summarized in the PRISMA flow diagram (Figure 1).

### 3.2. Study Characteristics and Salivary-Gland Analytical Context

The 25 included studies were published between 2015 and 2026, with 21 appearing from 2023 onward [8,10,11,12,13,14,28,29,30,31,32,33,34,35,36,37,38,39,40,41,42]. Twenty-three studies incorporated human data [8,9,10,11,12,25,26,27,28,29,30,31,32,33,34,35,36,37,38,39,40,41,42], whereas two were primarily controlled preclinical investigations [13,14]. By primary design, the corpus comprised 20 observational human tissue studies or secondary analyses of salivary-gland datasets [9,10,11,12,25,26,28,29,30,31,32,33,34,35,37,38,39,40,41,42], one randomized phase II trial with AI-assisted tissue-response analysis [27], two retrospective patient-level whole-slide-image studies [8,36], and two controlled preclinical intervention studies [13,14]. Twenty-one studies fulfilled the direct glandular Path A criterion [8,9,10,11,12,13,14,25,26,27,28,31,32,33,34,35,36,37,39,40,41], whereas four were retained through Path B or an integrative Path B route [29,30,38,42] because AI/ML discovery outside the gland was followed by substantial direct salivary-gland experimental, histopathological, or single-cell validation.

The analytical substrate was heterogeneous but consistently anchored to salivary-gland pathology. Five studies used histopathology or digital pathology as the primary analytical input [8,12,25,27,36], encompassing quantified fibrosis, serial treatment-response biopsies, multiplex spatial pathology, and whole-slide analysis of hematoxylin and eosin-stained tissue. Transcriptomic approaches predominated among molecular studies [26,28,31,32,33,34,35,37,39,41,42], alongside epigenomic [10,40], metabolomic or lipidomic [11,14], miRNA [13], and glandular single-cell analyses [38]; several of these studies incorporated orthogonal histological, immunohistochemical, immunofluorescence, animal, or cellular validation [13,14,30,31,35,37,38,39,42]. Using the methodological taxonomy defined in the eligibility criteria, the eligible computational approaches were grouped into five non-mutually exclusive classes. Conventional feature-selection/statistical-learning methods included penalized regression approaches such as LASSO or elastic net [11,13,30,31,32,33,34,35,37,38,39,41,42]. Classical machine-learning methods included random forests, support-vector machines, naïve Bayes classifiers, supervised cell-classification approaches, and gradient-boosting or decision-tree methods [11,12,14,25,26,27,28,29,30,31,32,33,34,35,37,38,39,41,42]. Unsupervised-learning approaches, principally hierarchical or consensus clustering, were used for data-driven phenotype or subgroup discovery [9,10,26,28,31,40]. Deep-learning approaches included variational autoencoders, convolutional neural networks, multiple-instance learning, and vision transformers [8,10,36,40]. Explainable-AI components included attention- or pattern-level attribution and SHAP/Shapley-based feature attribution [8,14,36]. Because several studies combined approaches from more than one class, these methodological categories were not mutually exclusive.

The geographic provenance of the human evidence was heterogeneous. The SICCA-based studies drew on an international resource established across Argentina, China, Denmark, India, Japan, the United Kingdom, and the United States [9,10,40]. Other directly recruited or independently validated cohorts included studies from the United States [25], France [11], Japan [12], and China [30,31,36,39,42]. Specifically, the independent glandular-validation cohorts in Zhou et al. [30] and Cai et al. [31] were recruited in Luoyang and Shanghai, respectively; Hou et al. [39] obtained their six SjD/six control salivary-gland samples at the First Hospital of Lanzhou University; and Yin et al. [42] recruited their independent validation cohort in Shandong Province. The multicenter digital-pathology study by Duquesne et al. [8] included six European expert centers in the United Kingdom, Greece, Portugal, and France, whereas the randomized treatment-response study by Mariette et al. [27] enrolled participants across 31 centers in 10 countries in Europe and the Americas. The principal recurrent public salivary-gland datasets also originated from a limited number of geographic settings: GSE23117 from the United States, GSE40611 from a Dutch cohort recruited at the University Medical Center Groningen, GSE127952 and GSE154926 from Brazil, GSE40568 and GSE80805 from Japan, and GSE173808/GSE208260 from the Netherlands; GSE7451, a whole-saliva dataset used in several cross-tissue analyses, originated from the United States. Repeated reuse of these public resources therefore reduced both the analytical and geographic independence of the evidence base. Study-level geographic provenance is summarized in Table 1.

Reuse of public datasets and cohorts was substantial. GSE23117, GSE40611, GSE127952, GSE154926, and GSE7451 recurred across multiple transcriptomic studies [26,28,31,32,33,34,35,39,41,42], while the Sjögren’s International Collaborative Clinical Alliance (SICCA) resource contributed overlapping or potentially overlapping participants to Hall et al. [9], Chi et al. [10], and Solomon et al. [40]. Accordingly, repeated identification of a gene, pathway, or subtype within shared resources was not interpreted as independent replication. Greater interpretive weight was assigned to findings reproduced in biologically independent cohorts, supported by orthogonal tissue modalities, or confirmed through direct experimental validation.

### 3.3. AI-Guided Histopathological Phenotyping

**Figure 2:**
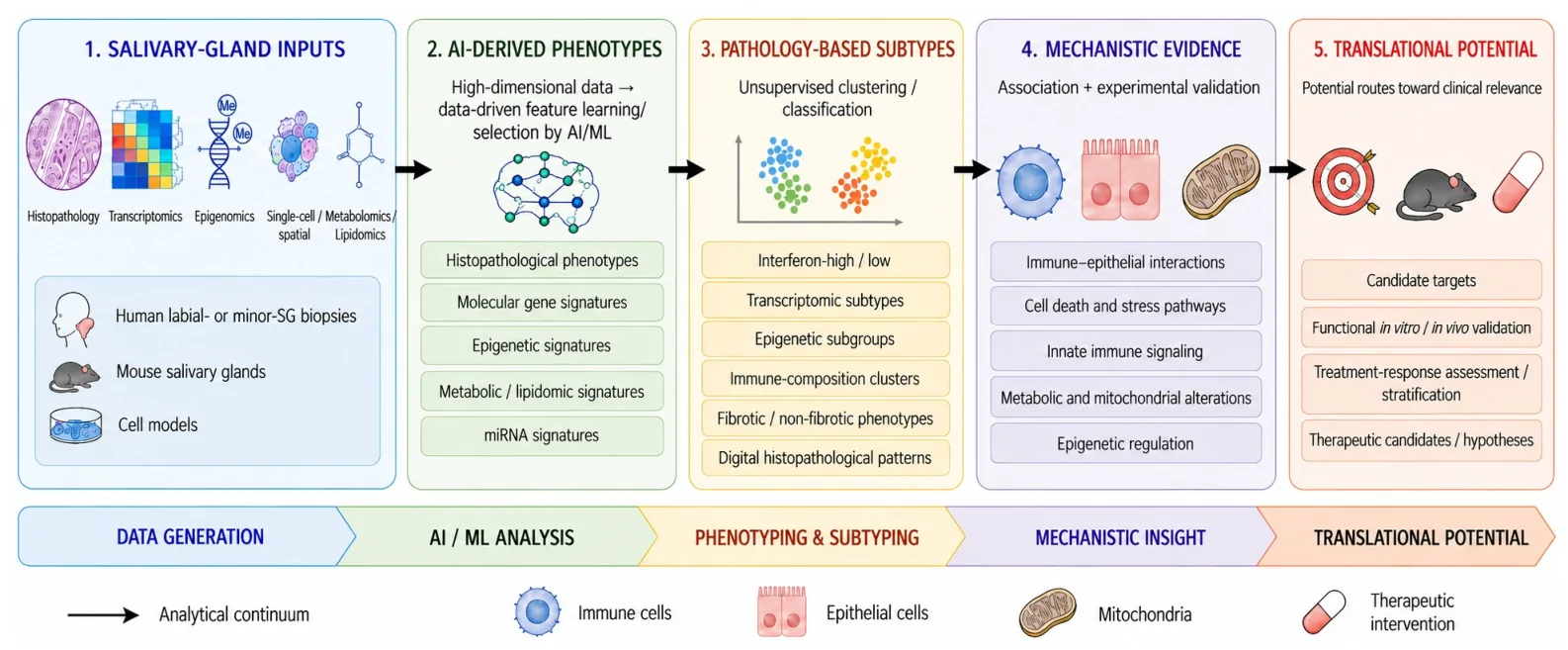
Functional landscape of AI applications in Sjögren’s salivary-gland pathology. The schematic organizes the included evidence from salivary-gland data inputs, including digital histopathology, transcriptomics, epigenomics, single-cell/spatial profiling, and metabolomics/lipidomics, through artificial intelligence and machine learning (AI/ML) analysis to histopathological and molecular phenotypes, pathology-based subtypes, mechanistic evidence, and potential translational extensions. The five color-coded panels represent salivary-gland inputs (blue), AI-derived phenotypes (green), pathology-based subtypes (yellow/orange), mechanistic evidence (purple), and translational potential (red/orange). The graphical symbols illustrate representative tissue and cellular models, immune cells (blue), epithelial cells (pink), mitochondria (brown), and potential therapeutic interventions (red-and-white capsule). Solid black arrows indicate the conceptual analytical continuum rather than obligatory transitions between stages. The figure represents a functional map of the evidence and does not imply that all studies progressed through every stage or that the sequence represents a mandatory developmental pathway. The final stage denotes translational potential rather than demonstrated clinical utility. Computational target or drug prioritization does not constitute evidence of therapeutic efficacy, and causality or treatment effects are inferred only where supported by direct experimental or interventional evidence.

AI-assisted tissue analysis identified histopathological patterns involving lymphocytic organization, fibrosis, epithelial injury, and spatial immune-cell relationships (Figure 2; Table 2).

Duquesne et al. [8] identified nine explainable histological patterns contributing to SjD classification, including lymphocytic foci, a vascular/fibrotic pattern, and a distinct periacinar lymphocytic pattern. Immunohistochemistry in additional biopsies characterized the periacinar cells as predominantly CD3+/CD8+, with partial CD103 expression. Ren et al. [36] localized WSI regions associated with prevalent high-risk extra-glandular organ involvement; pathological review of these regions identified lymphocytic infiltration, acinar or ductal injury/atrophy, and fibrosis.

Fibrosis and tissue spatial organization were evaluated with different computational approaches. In Leehan et al. [25], digitally quantified fibrosis was greater in SjD than in non-Sjögren sicca samples and remained associated with focus score after adjustment for age; the finding was reproduced across discovery and replication cohorts. Ono-Minagi et al. [12] generated epithelial, stromal, and immune-cell maps from multiplex-stained minor salivary-gland tissue and documented differences in epithelial alterations and immune-cell spatial relationships between primary and secondary SjD. Molecular autophagy findings from that study are reported under Mechanistic Insights.

### 3.4. Molecular and Multi-Omic Phenotyping

Across transcriptomic, metabolomic, single-cell, and related tissue-omic studies, AI/ML reduced high-dimensional salivary-gland data to smaller molecular signatures associated with interferon activity, programmed cell-death pathways, mitochondrial homeostasis, immune-cell composition, and glandular inflammation (Table 2; Figure 2). Transcriptomic feature-selection studies identified interferon/antiviral signatures [28,32], ferroptosis-related features [33], a TDP-43-associated signature [34], and pyroptosis- and mitophagy-related profiles [35,39]. Individual gene sets and their validation are summarized in Table 2.

Direct tissue metabolomics identified a six-metabolite labial-gland signature in Urbanski et al. [11]; its phospholipid component was inversely associated with lymphocytic sialadenitis grade, whereas kynurenine was associated with anti-SSA positivity. Single-cell analysis in Chao et al. [38] localized the selected molecular signal to monocyte/macrophage states and reconstructed a monocyte-to-macrophage trajectory in salivary-gland tissue. Yin et al. [42] combined consensus feature selection with an independent human glandular H&E/IHC cohort. Functional perturbation findings from Chao et al. and other molecular studies are reported separately under Mechanistic Insights.

### 3.5. Pathology-Based Subtyping

Unsupervised analyses identified glandular subgroups based on interferon activity, transcriptomic pathway profiles, and DNA methylation (Table 2; Figure 2). Hall et al. [9] separated SjD into IFN-low and IFN-high states and further resolved type I-predominant, type II-predominant, and mixed IFN-high patterns; focus score differed across these molecular groups. Min et al. [26] identified two transcriptomic/pathway clusters, with the higher-inflammatory cluster containing greater immune-cell enrichment and a higher proportion of moderate-to-advanced glandular inflammation.

Epigenetic analyses also identified distinct tissue subgroups. Chi et al. [10] separated labial-gland methylation profiles into clinically severe and mild SjD groups, whereas Solomon et al. [40] identified multiple methylation clusters in the larger SICCA cohort; composite subgroups differed in focus score, immune/epithelial cell proportions, and germinal-center frequency. Cai et al. [31] identified two molecular patterns with different interferon and immune states, and Wu et al. [41] identified two SjD molecular subtypes with different immune-infiltration profiles. Treatment-response associations and mechanistic outputs derived from these subgroup analyses are reported in subsequent sections.

### 3.6. Mechanistic Insights

Mechanistic studies addressed autophagy/mitophagy, programmed cell death, innate immune signaling, macrophage activation, epithelial injury, and metabolic stress (Table 2; Figure 2). Ono-Minagi et al. [12] reported increased glandular transcripts of *ATG5*, *LC3B*, and *SQSTM1* in secondary versus primary SjD, with LC3B and SQSTM1 proteins localized within lymphocytic infiltrates. Hou et al. [39] identified reduced expression of the mitophagy-related genes *PINK1*, *GABARAPL1*, and *SQSTM1*, accompanied by associations with glandular immune-cell composition. Dai et al. [35] demonstrated increased NLRP3, PECAM1, IFI16, AIM2, and MPEG1 proteins in inflamed salivary glands of NOD mice and confirmed increased expression of selected proteins in human labial-gland biopsies.

Innate immune and cell-death pathways were examined through direct glandular or functional validation. Wu et al. [29] provided in vivo glandular pathological anchoring of the systemically prioritized IFIH1 signal in the NOD model. IFIH1 expression was increased directly in damaged submaxillary glands, while H&E examination demonstrated lymphocytic infiltration of glandular tissue in the same disease model. This evidence was interpreted as preclinical glandular confirmation rather than independent human tissue validation or functional demonstration of a causal role for IFIH1. Wang et al. [37] linked the anoikis-related profile to glandular inflammation, fibrosis, epithelial apoptosis, and MAPK3 activation; pharmacological MAPK3 inhibition reduced apoptosis and inflammatory readouts in the epithelial-cell model. Chao et al. [38] localized *ISG15* to inflammatory monocyte/macrophage states in salivary-gland single-cell data, and *ISG15* knockdown reduced TNF-α and IL-1β expression and secretion in macrophages.

Two studies extended functional validation to defined regulatory or metabolic axes. Cai et al. [13] confirmed direct targeting of *CHUK* by miR-23b-3p and showed modulation of the IKKα/NF-κB pathway, oxidative stress, inflammatory cytokines, apoptosis, and the Treg/Th17 balance; re-expression of IKKα partially reversed these effects. Cui et al. [14] identified sphinganine binding to Myh9 and documented epithelial stress and fibrotic responses, whereas pharmacological Myh9 inhibition restored AQP5 expression and reduced fibrotic markers.

### 3.7. Validation Depth and Translational Implications

**Figure 3:**
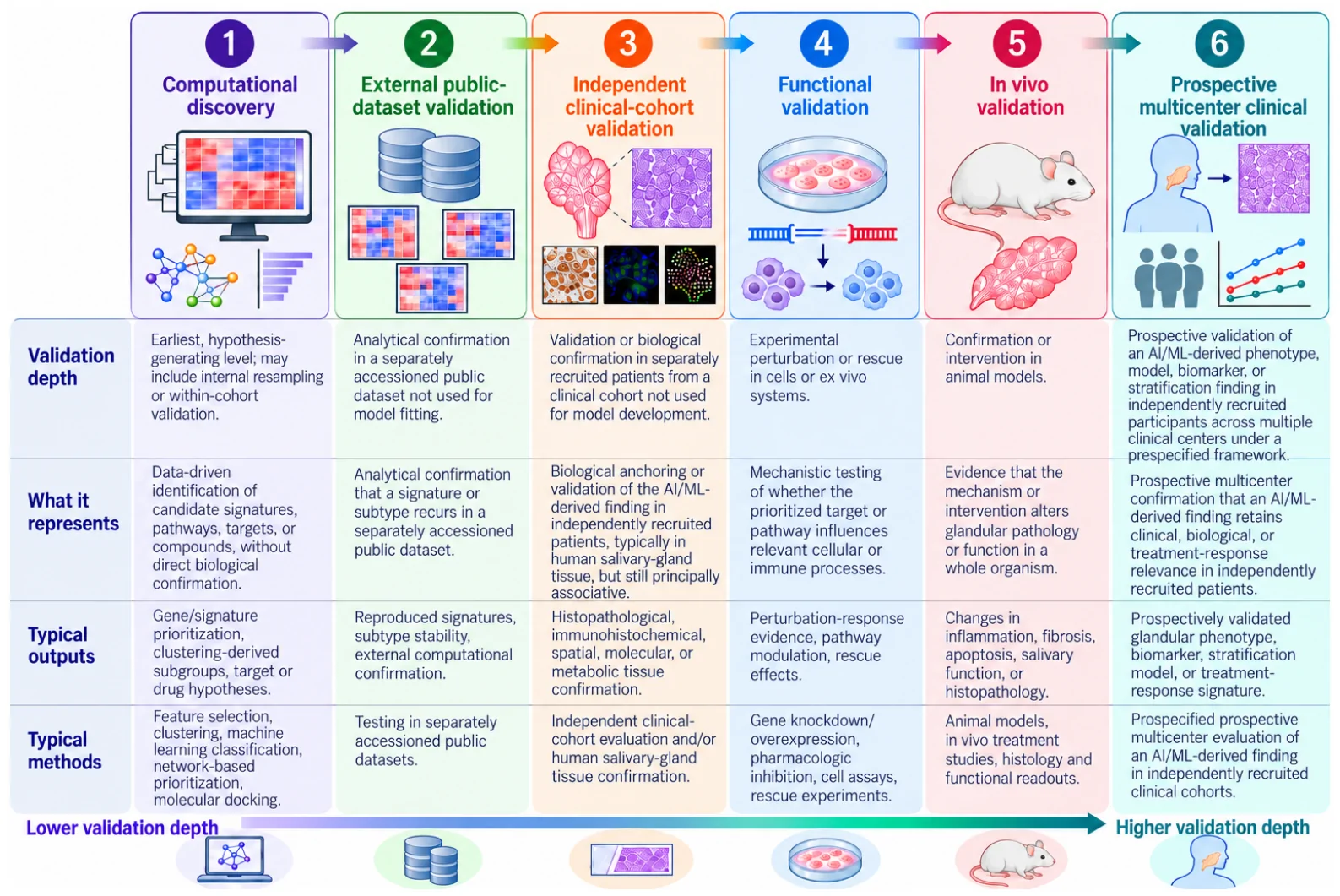
Validation-depth hierarchy of AI/ML-derived findings in Sjögren’s salivary-gland pathology. The diagram distinguishes six progressively deeper levels of validation, identified by different background colors: computational discovery (purple), external public-dataset validation (green), independent clinical-cohort validation (orange), functional validation through direct perturbation or rescue (blue), in vivo validation in animal models (pink), and prospective multicenter clinical validation (turquoise). The graphical symbols represent computational analyses and molecular signatures, independent public datasets, human tissue and clinical cohorts, experimental cell systems, animal models, and prospective multicenter clinical evaluation, respectively. The upper arrows indicate the conceptual progression between validation levels, while the lower gradient arrow represents increasing validation depth from left to right. These arrows do not imply that every study must progress sequentially through all levels. Internal resampling, cross-validation, or within-cohort data splitting was considered part of computational discovery and was not regarded as external validation. External public-dataset validation required confirmation in a separately accessioned public dataset not used for model fitting and without known participant overlap. Independent clinical-cohort validation required validation or biological confirmation in separately recruited human participants not used for model development. Functional validation required direct experimental perturbation or rescue, whereas in vivo validation required confirmation or intervention in an animal model. Prospective multicenter clinical validation required prespecified prospective evaluation of an AI/ML-derived phenotype, model, biomarker, or stratification finding in independently recruited participants across multiple clinical centers. Representative evidence ranged from predominantly computational or public-dataset-supported findings [26,28,32,33,34,41], through independent clinical-cohort validation [8,25,30,31,36,42], to functional validation [38] and in vivo validation [13,14,29,35,37]. Mariette et al. [27] provided prospective multicenter human treatment-response pathology with ML-assisted tissue quantification, but the AI/ML component served as an analytical measurement tool rather than as a prospectively validated AI-derived phenotype, model, biomarker, or stratification finding. It was therefore not classified as level 6. No included study reached prospective multicenter clinical validation as defined in this hierarchy. Placement within the hierarchy reflects validation depth and does not constitute a risk-of-bias judgment, formal certainty-of-evidence rating, or numerical quality score.

Validation depth varied markedly across the included evidence and was therefore synthesized according to the six-level hierarchy defined in Methods (Figure 3).

Several molecular studies remained at the computational-discovery or independent-dataset-validation levels, particularly when signatures were derived from secondary public datasets and reproduced predominantly through additional computational analyses [26,28,32,33,34,41]. Such replication was interpreted as analytical convergence rather than as independent biological confirmation.

A larger group of findings reached direct or independent human salivary-gland tissue validation. These studies linked AI/ML-derived phenotypes to histopathological, immunohistochemical, spatial, molecular, or metabolic findings in human glandular tissue, including fibrosis, interferon states, immunolipidomic profiles, spatial immune-cell relationships, molecular subtypes, and independently validated whole-slide phenotypes [8,9,10,11,12,25,30,31,36,39,42]. This level provided stronger biological anchoring than computational replication alone but did not, by itself, establish causality.

Functional validation was achieved when the prioritized molecular mechanism was directly perturbed in cellular or ex vivo systems. Chao et al. [38] showed that ISG15 knockdown modified macrophage inflammatory outputs, whereas Wang et al. [37] tested pharmacological MAPK3 inhibition in a salivary epithelial-cell model. Evidence progressed further to in vivo validation in studies incorporating direct animal-model confirmation or intervention [13,14,29,35,37]. Cai et al. [13] demonstrated therapeutic effects of SGEC-derived exosomal miR-23b-3p in NOD/Ltj mice, while Cui et al. [14] combined sphinganine perturbation with pharmacological Myh9 inhibition and documented functional and pathological rescue.

No included study reached level 6 prospective multicenter clinical validation as defined in the revised hierarchy. Mariette et al. [27] provided prospective multicenter human treatment-response pathology within a randomized phase II trial, in which serial minor-salivary-gland biopsies were analyzed using ML-assisted tissue quantification. However, the AI/ML component functioned as an analytical measurement tool for treatment-associated tissue changes rather than as an AI-derived phenotype, predictive model, biomarker, or stratification finding undergoing prospective multicenter validation. Accordingly, this study was not classified as level 6. Human treatment-response associations were also reported by Min et al. [26], but these findings likewise did not constitute prospective multicenter validation of an AI/ML-derived glandular phenotype. Overall, the evidence base remained weighted toward computational discovery, public-dataset validation, independent clinical-cohort confirmation, and selected functional or in vivo validation, with no study reaching the highest validation level.

### 3.8. Risk of Bias and Methodological Quality

**Figure 4:**
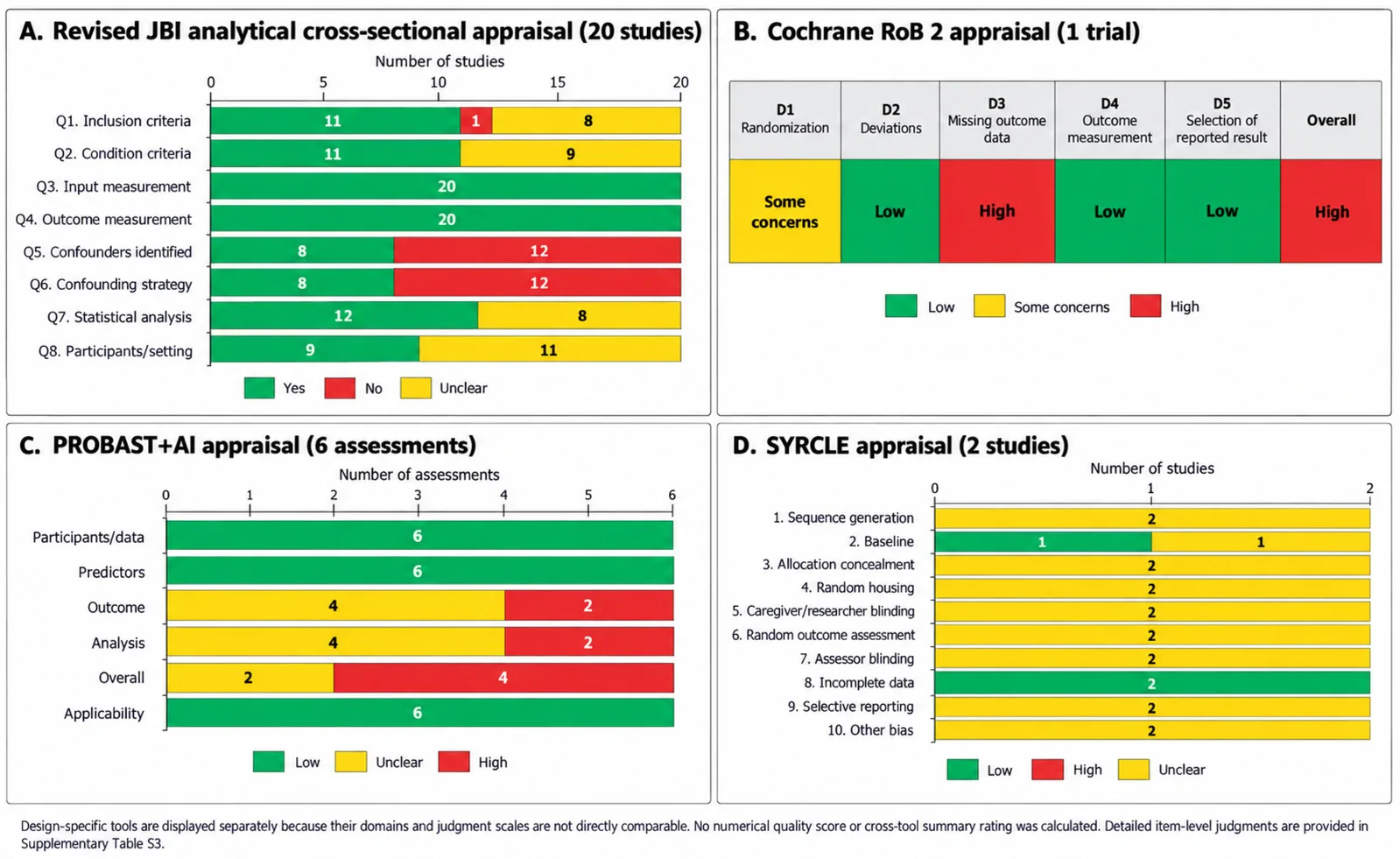
Design-specific summary of risk-of-bias and methodological-quality assessments. The four appraisal instruments are presented separately because their domains and judgment scales are not directly comparable. (**A**) Revised Joanna Briggs Institute (JBI) analytical cross-sectional appraisal of 20 studies [9,10,11,12,25,26,28,29,30,31,32,33,34,35,37,38,39,40,41,42], summarized as the number of Yes, No, and Unclear judgments for each of the eight items. (**B**) Cochrane Risk of Bias 2 (RoB 2) assessment of the randomized trial [27], showing the five domain-level judgments and the overall judgment. (**C**) Prediction model Risk Of Bias ASsessment Tool + AI (PROBAST + AI) appraisal of the participant-level prediction-model studies [8,36], summarized across the development and evaluation assessments as Low, Unclear, or High judgments for each domain; applicability is presented separately. (**D**) Systematic Review Centre for Laboratory animal Experimentation (SYRCLE) assessment of the controlled animal intervention studies [13,14], summarized as the number of Low, High, and Unclear judgments for each domain. No numerical quality score or cross-tool summary rating was calculated. Detailed study-level judgments and supporting rationale are provided in Supplementary Table S3.

Risk of bias and methodological quality were assessed according to study design using RoB 2, PROBAST + AI, the revised JBI analytical cross-sectional tool, or SYRCLE, as applicable (Supplementary Table S3; Figure 4). Of the 25 included studies, one randomized trial was assessed with RoB 2 [27]; two participant-level AI prediction studies with PROBAST + AI [8,36]; two controlled animal intervention studies with SYRCLE [13,14]; and the remaining 20 human tissue, molecular, or secondary salivary-gland dataset studies with the revised JBI analytical cross-sectional tool. Accordingly, the domains assessed and potential sources of bias differed according to study design. A design-specific visual summary is provided in Figure 4, with the four appraisal instruments displayed separately because their domains and judgment scales are not directly comparable.

Among the design-specific assessments, the randomized tissue-response outcome in Mariette et al. [27] was judged at high risk of bias, primarily because paired biopsy data were available for 60 of the 86 randomized participants. PROBAST + AI assessments of Duquesne et al. [8] ranged from unclear to high concern/risk of bias depending on the prediction task and development or evaluation component, whereas the Ren et al. [36] model was judged at high risk of bias for both development and evaluation. Principal concerns included incorporation of biopsy-derived reference information, high-dimensional analysis relative to sample size, incomplete reporting of data splitting, and absence of calibration. In the two controlled animal studies [13,14], most SYRCLE domains were judged unclear because sequence generation, allocation concealment, random housing, and blinding procedures were incompletely reported. Six of the twenty JBI-appraised studies met all eight assessed items [9,10,11,25,40,42], whereas incomplete control of confounding and limited characterization of source cohorts were recurrent concerns among several of the remaining studies (Supplementary Table S3).

The parallel AI/ML methodological-safeguards assessment identified several recurrent cross-study limitations that were not fully captured by the design-specific risk-of-bias tools (Supplementary Table S4). Leakage and analytical-independence safeguards were clearly satisfied in only 6 of 25 studies, whereas they were partially satisfied in 16, not satisfied in two, and considered not applicable in one study. Independent external validation was clearly demonstrated in 11 studies, partially demonstrated in five, and absent in eight; one study was not applicable to this criterion. Calibration was particularly weak: among the 16 studies for which it was considered applicable, none clearly satisfied this safeguard, with 15 not reporting or performing calibration and one providing only partial information. Complete data/code accessibility was also uncommon, being clearly satisfied in only two studies, partially satisfied in 22, and not satisfied in one. Biological or pathological validation was clearly demonstrated in 17 studies, partially in four, and absent in four. Internal validation or robustness procedures were clearly reported in 16 studies, partially reported in eight, and considered not applicable in one study. Across individual studies, additional concerns included high-dimensional analyses in relatively small samples, potential overfitting, incomplete separation of feature selection from model evaluation, and preprocessing or sample-selection procedures that could compromise strict independence between development and evaluation. These recurring safeguards were interpreted descriptively across the evidence base and were not combined into a cross-tool numerical quality score.

Validation setting also differed substantially across the evidence base. Internal resampling, cross-validation, or within-cohort data splitting was common, whereas much of the validation reported in molecular studies consisted of testing in additional public datasets. Such external public-dataset validation was distinguished from validation in independently recruited clinical cohorts and was not interpreted as equivalent evidence. Independent clinical-cohort validation was less frequent and was identified in selected histopathological or human tissue-validation studies [8,25,30,31,36,42]. Prospective multicenter validation of an AI-derived glandular phenotype or treatment-selection model was not demonstrated in the included evidence. Although Mariette et al. [27] provided prospective multicenter human treatment-response pathology with ML-assisted tissue quantification, this represented prospective clinical assessment of treatment-associated tissue changes rather than prospective validation of an AI-derived predictive model or glandular subtype. Accordingly, the strength of validation was interpreted according to the setting in which it was obtained rather than under a single generic category of external validation.

### 3.9. Reporting Bias and Confidence in the Evidence

Formal assessment of publication bias using funnel plots or statistical tests was not undertaken because the included studies did not provide a sufficiently homogeneous set of independent effect estimates for meta-analysis. Potential reporting bias was instead examined through comparison of stated study objectives; reported analytical procedures, supplementary information; and, when available, protocol or trial-registration information. No specific instance of selective omission of a prespecified primary analysis could be established; however, incomplete reporting of model-development procedures, calibration, unsuccessful or negative analyses, and analytical choices limited the ability to exclude selective reporting in several AI/ML studies.

Confidence in the synthesized evidence was evaluated narratively rather than through formal GRADE [23] ratings because the review integrated heterogeneous histopathological, molecular, computational, experimental, prediction-model, and treatment-response evidence rather than a single intervention- or diagnostic-effect question. Greater confidence was assigned to findings reproduced in biologically independent salivary-gland cohorts, corroborated across distinct analytical modalities, localized or confirmed directly in glandular tissue, or supported by functional perturbation. Conversely, confidence was more limited when findings depended primarily on secondary public datasets, repeated analysis of overlapping cohorts, internal computational validation, or in silico target or drug prioritization without independent biological confirmation. Dataset overlap was therefore considered explicitly when interpreting apparent replication across studies.

### 3.10. Structured Functional Synthesis

**Figure 5:**
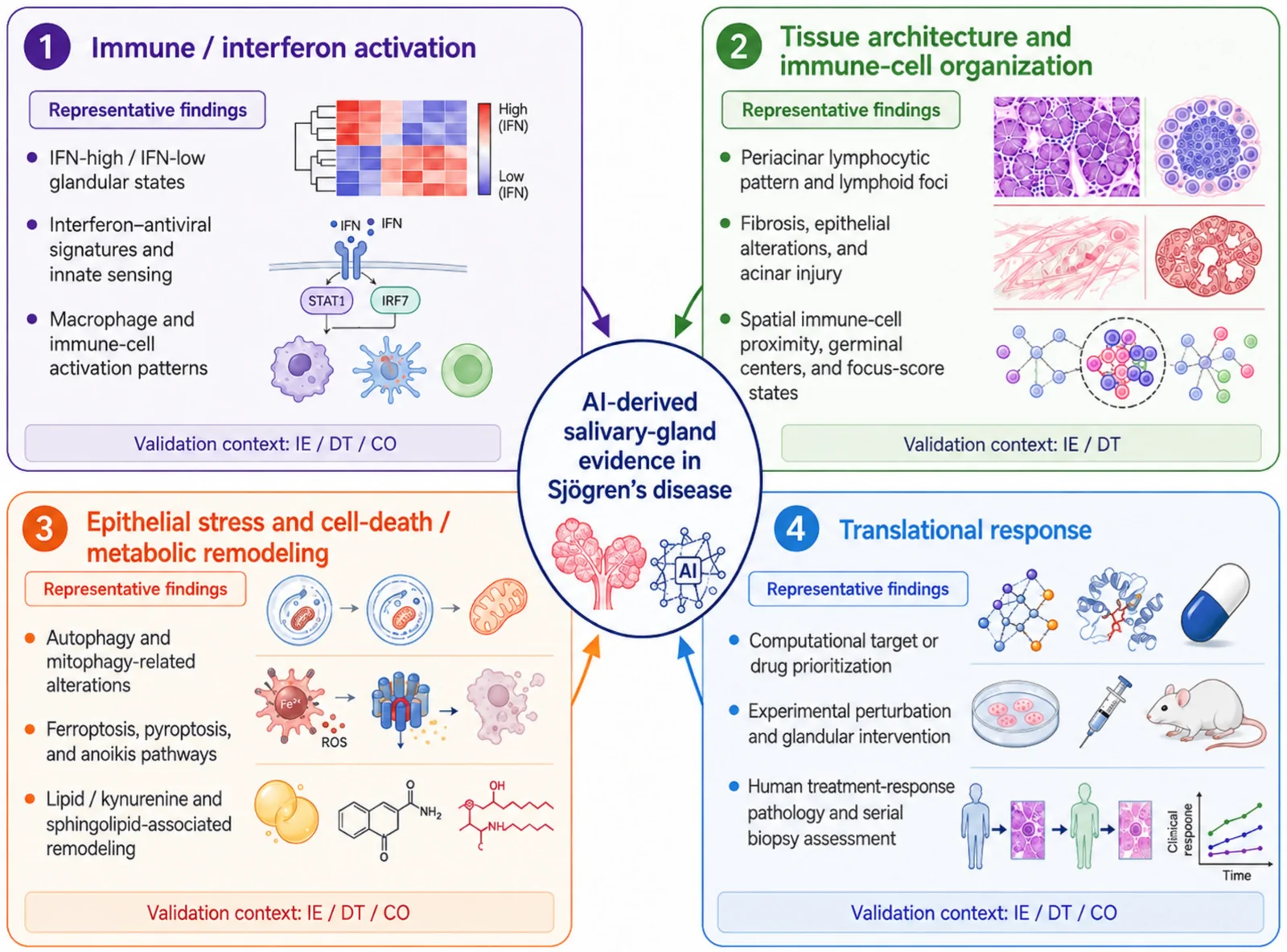
Structured functional synthesis of AI-derived salivary-gland evidence in Sjögren’s disease. The diagram integrates the included studies across four recurrent and non-mutually exclusive functional domains: immune/interferon activation (purple), tissue architecture and immune-cell organization (green), epithelial stress and cell-death/metabolic remodeling (orange), and translational response (blue). The graphical objects represent the principal findings within each domain, including interferon-related molecular signatures and immune-cell activation, glandular histopathology and immune-cell spatial organization, epithelial and mitochondrial injury, cell-death and metabolic pathways, computational target prioritization, experimental interventions, and human treatment-response assessment. The color-coded arrows connecting the four domains to the central circle indicate conceptual integration and functional convergence of AI-derived salivary-gland evidence rather than directional causality, temporal progression, or obligatory relationships between domains. AI-derived molecular and histopathological phenotypes are linked to their principal glandular pathological and functional correlates. Study references may contribute to more than one domain when an individual report provides evidence across multiple functional components. Validation context is displayed within each domain using the non-scored categories defined in the Methods and Table 2: IE, independent/experimental support based on validation in a separately recruited human clinical cohort and/or direct functional, experimental, or interventional validation; DT, direct or orthogonal glandular-tissue support without independent clinical-cohort or functional validation; and CO, predominantly computational convergence from secondary/public datasets without independent glandular biological confirmation. External public-dataset validation alone did not qualify as IE. For the immune/interferon activation domain, representative studies comprised IE [29,31,38], DT [9,40], and CO [26,28,32,41]. For tissue architecture and immune-cell organization, representative studies comprised IE [8,25,27,36] and DT [10,12,40]. For epithelial stress and cell-death/metabolic remodeling, representative studies comprised IE [13,14,35,37], DT [11,12,39], and CO [33]. For translational response, representative studies comprised IE [13,14,27,30,37,38,42], DT [9,39], and CO [26,32]. The validation-context categories are descriptive and do not represent study quality, formal certainty-of-evidence ratings, therapeutic efficacy, or pooled quantitative effects.

Across the 25 included studies, the evidence was functionally organized into four recurrent salivary-gland domains: immune/interferon activation, tissue architecture and immune-cell organization, epithelial stress and cell-death/metabolic pathways, and translational response (Figure 5).

Importantly, convergence within these functional domains did not represent equivalent levels of evidentiary support. Findings classified as independent/experimental support (IE) were supported by validation in a separately recruited human clinical cohort and/or by direct functional, experimental, or interventional validation of the AI/ML-derived finding [8,13,14,25,27,29,30,31,35,36,37,38,42]. External public-dataset validation alone did not qualify as IE. Other findings were anchored directly to salivary-gland tissue through histopathological, molecular, spatial, epigenetic, or small confirmatory tissue-series evidence but lacked independent clinical-cohort or functional validation and were therefore classified as direct tissue support (DT) [9,10,11,12,39,40]. Hou et al. [39] was included in this latter category because its additional glandular confirmation consisted of qRT-PCR in a small 6-SjD/6-control tissue series rather than independent clinical-cohort or functional validation. In contrast, several findings represented predominantly computational convergence (CO), often involving recurrent analysis of overlapping public GEO datasets without independent glandular biological confirmation [26,28,32,33,34,41]. Thus, recurrence of a molecular pathway or signature across studies was interpreted more cautiously when it reflected reuse of shared datasets rather than replication in biologically independent cohorts.

Immune and interferon-related phenotypes were identified across transcriptomic, epigenetic, and single-cell approaches. These included IFN-high and IFN-low glandular states [9], inflammatory transcriptomic clusters [26], interferon- and antiviral-related gene signatures [28,32], multi-tissue immune patterns [31], *IFIH1*-associated innate immune signaling [29], and *ISG15*-enriched macrophage states [38]. Across these studies, the AI-derived molecular phenotypes were linked to variation in glandular inflammatory activity, immune-cell composition, or histopathological severity.

A second evidence stream connected AI-derived phenotypes with the structural organization of salivary-gland pathology. Digital histopathology identified lymphocytic, periacinar, fibrotic, and tissue-injury patterns [8,25,36], whereas spatial and epigenetic analyses differentiated glandular states according to epithelial alterations, immune-cell proximity, focus score, germinal-center frequency, and immune/epithelial cellular composition [10,12,40]. These tissue-level findings converged with molecular studies of epithelial and metabolic stress. Ferroptosis-, pyroptosis-, anoikis-, autophagy/mitophagy-, and sphingolipid-related signatures were reported across glandular datasets and experimental models [12,14,33,35,37,39], while metabolomic profiling identified a glandular lipid/kynurenine phenotype associated with lymphocytic sialadenitis and anti-SSA status [11]. Functional perturbation was performed for selected pathways, including *ISG15*, MAPK3, the miR-23b-3p–IKKα/NF-κB axis, and the sphinganine–Myh9 axis [13,14,37,38].

The translational-response domain comprised computational target or drug prioritization [30,32,39,42], tissue-based stratification and treatment-response characterization [9,26,27], functional or pharmacological perturbation [14,37,38], and preclinical glandular intervention [13]. Across the four functional domains, individual studies frequently contributed evidence to more than one biological level, linking molecular signatures with glandular histopathology, immune-cell organization, experimentally modifiable pathways, or treatment-associated tissue changes. The structured functional synthesis therefore summarizes cross-study convergence among immune activation, tissue organization, epithelial/metabolic stress, and translational response without imposing a sequential or hierarchical relationship among these domains.

## 4. Discussion

This systematic review shows that AI/ML applications in SjD salivary-gland research have expanded beyond diagnostic classification toward multidimensional characterization of glandular pathology. Across the 25 included studies, computational approaches identified histopathological patterns, molecular and multi-omic signatures, and pathology-based subtypes that converged around four recurrent domains: immune/interferon activation, tissue architecture and immune-cell organization, epithelial stress and cell-death/metabolic remodeling, and translational response. Selected findings were localized or reproduced directly in salivary-gland tissue, whereas only a smaller subset progressed to functional perturbation, pharmacological testing, in vivo intervention, or assessment of treatment-associated changes in human glandular tissue. Accordingly, the present evidence supports the translational potential of AI/ML-derived glandular phenotypes rather than established translational utility. AI/ML may provide a framework for resolving biological heterogeneity within the affected gland and for generating mechanistically and clinically relevant hypotheses, but computational discovery still substantially exceeds independent biological validation, and evidence connecting AI-derived glandular phenotypes with clinically actionable treatment selection remains limited.

The six-level validation hierarchy further emphasizes the unequal inferential weight of these findings. Reproduction of a molecular signature across independent datasets increases analytical robustness but does not establish its biological relevance in glandular tissue, and direct human tissue confirmation remains principally associative. Stronger mechanistic inference requires experimental perturbation or rescue, followed by in vivo confirmation where appropriate. Prospective multicenter clinical validation is ultimately required to establish whether an AI/ML-derived glandular phenotype, biomarker, or stratification model retains biological or treatment-response relevance in independently recruited patients under clinical conditions. Accordingly, recurrent signatures derived predominantly from public datasets should not be interpreted at the same evidentiary level as pathways supported by functional perturbation, animal intervention, or prospectively collected human treatment-response evidence, which itself should be distinguished from prospective validation of an AI/ML-derived phenotype or model.

This perspective extends the trajectory established by previous reviews. Fisher et al. [5] had already emphasized that minor salivary-gland histopathology could provide prognostic, stratification, and treatment-response information beyond diagnosis, while highlighting limitations in reproducibility and tissue scoring. In 2018, Foulquier et al. [43] identified 12 Sjögren-related studies using advanced computational approaches but none specifically addressing pathogenesis-based treatment, whereas the broader review by Stafford et al. [44] found no eligible Sjögren study under its criteria and identified independent validation as a major limitation of autoimmune ML research. More recent reviews reflect the subsequent expansion of the field but remain predominantly diagnosis-oriented. Rawat et al. [15] synthesized AI applications across histopathology, imaging, genomics, metabolomics, and electronic health records primarily for improving SjD diagnosis. Chatzis and Tzioufas [45] subsequently positioned the salivary-gland biopsy as a platform for precision medicine, highlighting digital pathology, tissue pathotypes, multimodal integration, and therapeutic-target discovery, while Zhou et al. [46] identified the transition from correlative AI models toward mechanistically defined subtypes as an important future direction. The present review addresses this interface specifically from the salivary-gland pathology perspective by integrating histopathological and molecular phenotyping with subtyping, mechanistic validation, and translational evidence.

A major implication of this synthesis is that AI/ML may help resolve glandular heterogeneity that is incompletely represented by conventional histopathological indices. Focus score remains fundamental, but it reduces a spatially and cellularly heterogeneous inflammatory process to the number of lymphocytic foci per unit area. Features such as inflammatory burden, germinal-center organization, fibrosis, cellular composition, and spatial architecture may convey additional information [5,45]. In the included studies, digital pathology identified periacinar lymphocytic, fibrotic, tissue-injury, and spatial immune-cell patterns [8,12,25,36], whereas transcriptomic and epigenetic analyses distinguished interferon-defined, inflammatory, methylation, and immune-composition states [9,10,26,31,40,41]. Interferon activity emerged as the most recurrent molecular signal, encompassing IFN-high/low phenotypes, type I- and type II-predominant profiles, antiviral signatures, *IFIH1*-associated innate sensing, and *ISG15*-enriched macrophage states [9,28,29,31,32,38]. Together with periacinar CD8+ patterns, altered T-cell/B-cell proximity, germinal-center organization, and differences in epithelial and immune-cell composition [8,12,40], these findings support a view in which morphologic severity, molecular state, cellular identity, and spatial organization represent complementary dimensions of glandular pathology.

An important unresolved question is whether these AI/ML-derived subgroups represent stable biological endotypes or, at least in part, context-dependent disease states. Several of the identified patterns were associated with contemporaneous clinical or pathological characteristics: interferon-defined states differed in focus score [9], the higher-inflammatory transcriptomic cluster was associated with more advanced glandular pathology and differential rituximab response [26], methylation-derived groups separated clinically more severe from milder phenotypes [10], and the larger SICCA methylation analysis identified subgroups differing in focus score, immune/epithelial composition, and germinal-center frequency [40]. Other findings were related to anti-SSA status [11] or extra-glandular organ involvement [36], while fibrosis remained associated with focus score after adjustment for age in one study [25]. These observations indicate that some data-driven clusters may capture disease activity, tissue inflammatory burden, serological status, systemic involvement, treatment exposure, disease duration, age, or cohort-selection effects rather than invariant SjD endotypes. Importantly, these clinical covariates and the classification criteria used to define SjD were not uniformly reported, harmonized, or incorporated into the analytical models across studies. Cross-sectional clustering alone therefore cannot establish temporal stability or distinguish a persistent biological endotype from a disease-stage or treatment-related state. Prospective longitudinal studies should evaluate whether the same phenotypes remain stable over time and across treatment, independently of disease activity, anti-SSA/SSB status, systemic involvement, age, disease duration, and classification framework, before they are interpreted as clinically meaningful SjD endotypes.

The evidence also extends the SjD glandular model beyond lymphocytic infiltration toward epithelial stress, programmed cell death, mitochondrial homeostasis, and metabolic remodeling. Autophagy/mitophagy [12,39], ferroptosis [33], pyroptosis [35], anoikis-related pathways [37], sphingolipid-associated stress [14], and a tissue lipid/kynurenine phenotype [11] were identified across computational and experimental studies. These pathways should not be regarded as equally established mechanisms, because their evidential depth varied substantially. Computational feature importance, network centrality, or classification performance can prioritize biologically plausible candidates but cannot establish causality. Some studies progressed to direct glandular expression or protein confirmation [29,35,39,42], whereas others incorporated spatial or single-cell localization [12,38]. Only a smaller group performed functional perturbation: *ISG15* knockdown altered macrophage inflammatory outputs [38], MAPK3 inhibition reduced epithelial apoptosis and inflammatory mediators [37], manipulation of the miR-23b-3p–*CHUK*/IKKα/NF-κB axis produced reciprocal functional effects [13], and the sphinganine–Myh9 relationship was supported by target identification and pharmacological rescue [14]. Mechanistic interpretation should therefore remain proportional to the experimental level actually demonstrated.

Evidence relevant to translational potential has expanded since the absence of pathogenesis-based AI studies reported by Foulquier et al. [43], but it remains markedly uneven. Computational drug–gene matching, network pharmacology, and molecular docking are represented in several studies [30,32,39,42], although these analyses generally stop before experimental testing of the prioritized compounds. A smaller group progressed to pharmacological perturbation [14,37], preclinical glandular intervention [13], or analysis of treatment-associated molecular and histopathological changes in humans [26,27]. This distinction is critical because docking and database-based matching support candidate prioritization but do not constitute evidence of drug efficacy, validated repurposing, or clinical utility. Likewise, the association between glandular molecular states and rituximab response [26] and ML-assisted quantification of tissue changes during sequential belimumab/rituximab treatment [27] provide proof-of-concept that glandular phenotyping may have future relevance for therapeutic stratification, but they do not establish validated treatment-selection biomarkers. At present, AI-derived salivary-gland subtypes therefore remain investigational and should be interpreted as candidates for prospective validation rather than as clinically actionable phenotypes.

Methodological limitations remain a major barrier to that transition. Reuse of a relatively restricted set of public salivary-gland datasets means that recurrence of a molecular signature across publications does not necessarily represent independent replication. The present review therefore mapped dataset overlap and did not consider repeated use of GSE or SICCA cohorts as independent external confirmation. This problem accompanies broader weaknesses in validation, including small retrospective cohorts, incomplete separation of feature selection from performance estimation, limited calibration, and infrequent prospective or multicenter evaluation. Similar concerns were identified by Stafford et al. [44] and more recent SjD AI reviews [46]. Future studies should distinguish technical from biological replication, preserve truly independent external cohorts, and explicitly document cohort provenance and batch effects. Digital-pathology studies additionally require harmonization across staining protocols, scanners, annotations, and institutions. Importantly, simply increasing cohort size will not resolve these problems if the same patients, datasets, or analytical leakage remain embedded across development and validation stages.

A more consequential next step would be to integrate modalities within the same patients rather than continue producing isolated single-omic classifiers. Chatzis and Tzioufas [45] proposed integration of histopathology with immunohistochemistry, single-cell, spatial, and other omic information as a route toward tissue endotyping, while Zhou et al. [46] emphasized the need for multimodal and more mechanistically informed AI. The evidence synthesized here indicates that the necessary components already exist but remain fragmented: digital histopathology captures architecture and spatial organization; transcriptomics and epigenomics define molecular states; single-cell analyses assign signals to cellular compartments; metabolomics and lipidomics provide complementary functional information; and perturbation experiments test biological consequence. Their integration in prospectively characterized patients could provide a more rigorous basis for pathology-defined endotypes than retrospective linkage of signatures derived from unrelated public cohorts.

From a pathology perspective, AI-enabled analysis should therefore be viewed as complementary to, rather than a replacement for, conventional salivary-gland assessment. The relevant objective is not automation per se, but more reproducible quantification of inflammatory burden, fibrosis, epithelial injury, lymphoid organization, cellular composition, and spatial relationships, together with their molecular context. Such integration could eventually move biopsy interpretation from a predominantly morphology-based assessment toward a multidimensional pathological phenotype capable of supporting biological stratification and treatment-response research. Current evidence does not yet justify replacing expert histopathological interpretation or introducing AI-derived molecular subtypes into routine clinical decision-making. Prospective multicenter cohorts, standardized tissue sampling and pathological endpoints, independent validation, explicit management of dataset overlap, and integrated whole-slide, molecular, and spatial profiling are therefore priorities. Candidate pathways should progress from computational ranking to glandular confirmation and, where appropriate, perturbation and rescue before therapeutic claims are advanced, while treatment-response studies should determine whether AI-derived tissue phenotypes provide information beyond established clinical, serological, and conventional histopathological variables.

Several limitations of this review should also be acknowledged. The evidence was heterogeneous in study design, biological substrate, AI/ML methodology, outcome definition, and experimental depth, precluding conventional quantitative meta-analysis. Many molecular investigations relied on retrospective public datasets, and recurrent cohort reuse reduced the effective independence of the evidence base. Human tissue cohorts were generally limited in size, and independent clinical-cohort validation was uncommon, while prospective multicenter validation of an AI-derived glandular phenotype or treatment-selection model was not demonstrated. Publication bias toward positive AI/ML findings and selective reporting of the best-performing models or analytical pipelines cannot be excluded, particularly because unsuccessful or negative analyses were inconsistently reported. Small, high-dimensional datasets also increase the potential instability of feature selection and the risk of overfitting, while incomplete separation of preprocessing or feature selection from model evaluation may permit information leakage in some analyses. Batch effects arising from differences in platforms, laboratories, preprocessing pipelines, and public-dataset integration were not consistently characterized or controlled. Reproducibility was further limited by incomplete public availability of analytical code, trained models, or sufficiently detailed implementation information in many studies. Clinical determinants that could influence glandular molecular or histopathological profiles—including disease duration and activity, serological status, systemic involvement, prior treatment, age, and classification criteria—were also inconsistently characterized or incorporated into the analyses, limiting the ability to distinguish stable biological endotypes from context-dependent disease states.

Additional heterogeneity arose from the salivary-gland substrate itself. The included studies analyzed labial or other minor salivary glands, parotid tissue, and, in experimental studies, predominantly submandibular glands from animal models; findings derived from these anatomically and biologically distinct glands should not be assumed to be directly interchangeable. Tissue acquisition and processing also varied across studies, including biopsy sampling, fixation and preservation, histological preparation, molecular-platform procedures, and tissue-quality requirements. Moreover, differences in inflammatory burden, fibrosis, acinar or ductal preservation, and the relative proportions of epithelial, stromal, and immune-cell populations may materially influence both histopathological and molecular signatures. These sources of pre-analytical and tissue-composition heterogeneity were not uniformly measured or adjusted for and therefore limit direct cross-study comparison and the interpretation of apparently convergent AI/ML-derived phenotypes. Future studies should report gland type, tissue-processing procedures, biopsy adequacy, histopathological composition, and cellular proportions systematically and should validate candidate phenotypes across anatomically distinct salivary-gland compartments before assuming generalizability.

Although some studies drew on multinational resources, geographic generalizability remains uncertain because several independent cohorts were recruited within single countries and repeated use of SICCA and GEO-derived samples does not constitute independent validation across populations, ancestries, or healthcare settings. Combining observational human studies, secondary molecular datasets, experimental models, and intervention studies was necessary to trace the continuum from phenotype discovery to mechanistic and translational validation, but these evidence types do not carry equivalent inferential weight. More broadly, the predominantly retrospective, exploratory, and research-oriented nature of the evidence limits generalizability to routine clinical practice, where patient populations, tissue acquisition, laboratory workflows, scanner or platform characteristics, and available computational infrastructure may differ substantially from those represented in the included studies. In addition, this review deliberately excluded AI applications restricted to diagnosis or focus-score automation unless they provided eligible biological, pathological, mechanistic, or treatment-stratification information. Its conclusions therefore apply specifically to AI-enabled salivary-gland phenotyping and pathobiology rather than to the entire diagnostic AI literature in SjD.

## 5. Conclusions

AI/ML is expanding the characterization of salivary-gland pathology in SjD beyond diagnostic classification by enabling histopathological and molecular phenotyping, pathology-based subtyping, and investigation of candidate disease mechanisms. Across the available evidence, recurrent findings converged around immune/interferon activation, tissue architecture and immune-cell organization, epithelial stress and cell-death/metabolic remodeling, and translational response. However, most findings remained at the computational, dataset-validation, or tissue-confirmation levels, and only a limited subset progressed to functional perturbation, in vivo validation, or prospective human treatment-response assessment.

The current evidence therefore supports translational potential rather than demonstrated translational utility. AI-derived glandular phenotypes cannot yet be considered validated biomarkers for treatment selection or routine therapeutic decision-making. Recurrent public-dataset use, limited independent validation, small tissue cohorts, heterogeneous analytical approaches, and scarce prospective clinical validation remain major barriers. Future progress will require prospective multicenter studies integrating digital histopathology with molecular, single-cell, and spatial data in the same patients, followed by independent biological, functional, and clinical validation before AI-assisted glandular phenotyping can contribute reliably to precision pathology or therapeutic stratification in SjD.

## Figures and Tables

**Table 1:** Characteristics and primary analytical role and geographic provenance of the included studies.

Study	Design/Evidence Source	Salivary-Gland Substrate	AI/ML Approach	Primary Pathology-Oriented Role	Geographic Provenance
Hall et al., 2015 [9]	Human cross-sectional; SICCA (53 SjD/29 controls)	Frozen labial SG; tissue IFN markers	Hierarchical clustering; multivariable modeling	Molecular phenotyping/IFN-based subtyping	SICCA international cohort: Argentina, China, Denmark, India, Japan, United Kingdom, and United States.
Leehan et al., 2018 [25]	Human discovery/replication cohorts	Minor labial SG H&E; quantified fibrosis	VSURF/random-forest variable selection	Histopathological phenotype discovery and replication	United States (direct discovery/replication cohorts).
Min et al., 2019 [26]	Secondary SG transcriptomic integration; rituximab-response subset	GSE7307, GSE23117, GSE40611, GSE80805	Hierarchical clustering; naïve Bayes; Bayesian key-driver analysis	Molecular subtyping, mechanisms and response stratification	Secondary public datasets with multi-country provenance; treatment-response subset reported separately in the source study.
Mariette et al., 2022 [27]	Randomized phase II trial; n = 86	Serial minor-SG H&E/IF; CD20/CD138	Definiens ML pattern recognition for tissue/cell segmentation	AI-assisted treatment-response histopathology	Multinational: 31 centers in 10 countries across Europe and the Americas.
Cerdó et al., 2024 [28]	Secondary human omics study	GSE23117 SG + GSE7451 saliva	Random forest; clustering	Glandular molecular feature prioritization	United States-derived public datasets (GSE23117 and GSE7451).
Chi et al., 2023 [10]	Human SICCA epigenetic study; n = 131	Labial SG DNA methylation	Variational autoencoder + hierarchical clustering	Epigenetic phenotyping/subgroup discovery	SICCA international cohort: Argentina, China, Denmark, India, Japan, United Kingdom, and United States.
Urbanski et al., 2023 [11]	Prospective human cohort; 40 pSS/40 sicca controls	Direct labial-SG targeted metabolomics	Elastic-net logistic regression + random forest	Immunometabolic phenotype linked to sialadenitis	France (prospectively recruited clinical cohort).
Wu et al., 2023 [29]	Systemic human discovery + mouse validation	Blood/PBMC discovery; NOD submandibular SG validation	WGCNA/PPI + SVM	Path B target discovery with direct glandular validation	China (Nanjing clinical and mouse validation); systemic discovery used public blood datasets.
Zhou et al., 2023 [30]	Human ML discovery + independent pathology cohort	Blood discovery; 85 pSS/20 normal LSG H&E/IHC	SVM, LASSO, RF, WGCNA	Target prioritization with human glandular pathology validation	China (Luoyang; independent 85 pSS/20 control labial-gland pathology cohort).
Cai et al., 2023 [31]	Integrative multi-tissue human study + LSG IHC	Multiple GEO datasets including SG; independent LSG IHC	WGCNA, LASSO, RF; consensus clustering	Cross-tissue phenotyping/tissue subtyping	China (Shanghai; independent 7 pSS/4 control labial-gland IHC cohort); secondary GEO datasets from multiple source populations.
Liu et al., 2024 [32]	Secondary human SG transcriptomic study	GSE40568/GSE40611; GSE127952/GSE154926	WGCNA + LASSO/SVM-RFE/RF	SG molecular target discovery	Public GEO source populations: Japan, Netherlands, and Brazil.
Ono-Minagi et al., 2024 [12]	Human retrospective spatial pathology	Minor-SG H&E, nCounter, multiplex IF	QuPath + supervised ML cell classification	Digital/spatial histopathological phenotyping	Japan (direct human salivary-gland cohort).
Yang et al., 2024 [33]	Secondary human SG transcriptomic study	Multiple parotid/minor-SG GEO datasets	LASSO + random forest	Ferroptosis-related molecular phenotyping	Secondary public salivary-gland datasets with multi-country provenance (principal recurrent sources summarized below).
Zhang et al., 2024 [34]	Secondary human SG/saliva molecular study	GSE7451, GSE23117, GSE40611	LASSO + SVM-RFE	Candidate mediator discovery	Public GEO source populations: United States and Netherlands.
Dai et al., 2025 [35]	Human SG ML discovery + mouse/human-tissue validation	GSE40611/GSE127952; GSE154926; NOD/human SG	LASSO + SVM-RFE	Pyroptosis-related phenotype and target discovery	China (human/animal experimental validation); public GEO source populations: Netherlands and Brazil.
Duquesne et al., 2025 [8]	Multicenter retrospective WSI study; n = 545	Digitised H&E minor-SG biopsies; external-centre validation	CLAM MIL; ViT; NMF/Shapley explainability	Explainable histopathological phenotype discovery	United Kingdom, Greece, Portugal, and France (six European expert centers).
Ren et al., 2025 [36]	Retrospective WSI study; n = 221	Labial-SG whole-slide images	CNN backbones + ViT/MIL ensemble	Histopathologic stratification of prevalent HR-OI	China (development and external clinical cohorts).
Wang et al., 2025 [37]	Human molecular study + mouse/cell validation	GEO SG data; mouse submandibular SG; SG epithelial cells	WGCNA + LASSO/SVM-RFE/RF	Anoikis-related phenotype and functional target testing	China (mouse/cell experimental validation); secondary public datasets with multi-country provenance.
Cai et al., 2026 [13]	Preclinical discovery-to-intervention study	Mouse SG miRNA profiling; SGECs; SG histology/IHC	WGCNA + LASSO	miRNA mechanism discovery + in vivo therapeutic validation	Preclinical study; no independently recruited human cohort.
Chao et al., 2026 [38]	Integrative systemic-to-glandular human study	Blood discovery; LSG scRNA-seq; THP-1 validation	WGCNA + LASSO/SVM-RFE/RF; pseudotime/CellChat	Integrative macrophage/*ISG15* mechanism discovery	Secondary human transcriptomic/single-cell datasets; functional cell validation performed in China.
Cui et al., 2026 [14]	Preclinical gland/lung lipidomics + intervention	Submandibular SG and lung lipidomics	Logistic regression + RF + SHAP	Metabolite mechanism discovery + causal perturbation/rescue	Preclinical study; no independently recruited human cohort.
Hou et al., 2026 [39]	Secondary human SG transcriptomics + own tissue qRT-PCR	GSE23117/GSE40611; GSE173808; 6/6 SG tissues	LASSO + random forest	Mitophagy-related molecular phenotyping	China (Lanzhou; own 6 SjD/6 control SG tissue series); public GEO source populations: United States and Netherlands.
Solomon et al., 2026 [40]	Large human SICCA methylation study; n = 1059	Labial-SG EPIC methylation	VAE + consensus/hierarchical clustering	Large-scale epigenetic subgroup discovery	SICCA international cohort: Argentina, China, Denmark, India, Japan, United Kingdom, and United States.
Wu et al., 2026 [41]	Cross-disease secondary transcriptomic study	SjD GSE40611/GSE127952 + MALT/thyroid cohorts	WGCNA; SVM/RF/LASSO; ssGSEA/CIBERSORT	Cross-disease molecular/immune stratification	Secondary public datasets with multi-country provenance; SjD source populations include Netherlands and Brazil.
Yin et al., 2026 [42]	Mixed human omics + own tissue validation	Multi-tissue GEO data; own parotid H&E/IHC	DT, XGBoost, RF, LASSO, GBM	Consensus biomarkers with direct glandular pathology validation	China (Shandong; independent 10 SjD/10 control validation cohort); secondary GEO datasets from multiple source populations.

Abbreviations: AI, artificial intelligence; GBM, gradient boosting machine; H&E, hematoxylin and eosin; HR-OI, high-risk extra-glandular organ involvement; IHC, immunohistochemistry; IF, immunofluorescence; LSG, labial salivary gland; MIL, multiple-instance learning; NMF, non-negative matrix factorization; RF, random forest; SG, salivary gland; SjD, Sjögren’s disease; SVM-RFE, support vector machine–recursive feature elimination; VAE, variational autoencoder; ViT, vision transformer; WGCNA, weighted gene co-expression network analysis. Geographic provenance refers to the source population or recruitment site of the biological material rather than the authors’ institutional affiliation. Principal recurrent public datasets originated from GSE23117 and GSE7451 (United States), GSE40611 and GSE173808/GSE208260 (Netherlands), GSE40568 and GSE80805 (Japan), GSE127952 and GSE154926 (Brazil), GSE51092 (United States), GSE66795 (United Kingdom), and GSE84844 (Japan). Where studies integrated multiple public datasets, the countries shown reflect source-population provenance rather than the location of the secondary analysis.

**Table 2:** AI-derived salivary-gland phenotypes, pathological correlates, and validation context.

Study	AI-Derived Phenotype/ Signature	Salivary-Gland Pathological Correlate	Validation/Replication
**AI-guided histopathological phenotyping**			
Leehan et al., 2018 [25]	Quantified minor-salivary-gland fibrosis phenotype.	Fibrotic area was greater in SjD and associated with focus score independently of age.	(IE) Independent clinical-cohort replication: human discovery and replication cohorts with digital H&E-based fibrosis quantification.
Ono-Minagi et al., 2024 [12]	Spatial autophagy-related epithelial, stromal, and immune-cell states.	LC3B/SQSTM1-positive regions and altered immune-cell spatial relationships within lymphocytic infiltrates.	(DT) No separate external validation. Direct human FFPE tissue with digital RNA, multiplex immunofluorescence, and supervised cell classification.
Duquesne et al., 2025 [8]	Explainable WSI patterns, including a periacinar lymphocytic phenotype.	CD3+/CD8+ (partly CD103+) lymphocytes around structurally preserved acini; additional focus/fibrosis patterns.	(IE) Independent clinical-cohort validation (multicenter, retrospective) plus independent immunohistochemical characterization of the AI-derived pattern.
Ren et al., 2025 [36]	WSI phenotype associated with prevalent high-risk extra-glandular organ involvement.	Lymphocytic infiltration, acinar/ductal injury or atrophy, and fibrotic changes in high-risk tissue regions.	(IE) Internal validation followed by independent clinical-cohort validation at an external center, with pathological review of attention regions.
**Molecular and multi-omic phenotyping**			
Cerdó et al., 2024 [28]	Shared salivary-gland/saliva antiviral-interferon gene signature including *CARD16*, *CMPK2*, *IFIT2*, *IFIT3*, and *RSAD2*.	Glandular transcriptomic inflammatory phenotype; no new morphology.	(CO) Random-forest prioritization; no independent tissue experiment. No independent clinical-cohort validation was reported.
Urbanski et al., 2023 [11]	Six-metabolite labial-gland immunolipidomic phenotype (kynurenine plus five phospholipids).	Phospholipid component inversely associated with lymphocytic sialadenitis grade; kynurenine associated with anti-SSA.	(DT) Internal validation: prospective direct LSG metabolomics with train/validation/test analysis.
Liu et al., 2024 [32]	*SAMD9*/*IFIT3*-centered glandular transcriptomic phenotype.	No independent morphology; immune-infiltration associations derived from glandular expression data.	(CO) External public-dataset validation; no independent experimental glandular validation.
Yang et al., 2024 [33]	Ferroptosis-related glandular signature: *TBK1*, *SLC1A4*, *PIK3CA*, *ENO3*, *EGR1*, and *ATG5*.	Transcriptomic salivary-gland phenotype with immune-cell estimates.	(CO) Internal/algorithmic validation after integration of multiple salivary-gland public datasets; no independent clinical-cohort or tissue validation.
Zhang et al., 2024 [34]	TDP-43-associated feature set including *CSF2RB*, *TRIM22*, *VAMP7*, *IFIT1*, *C21orf2*, and *PPM1H*.	Glandular expression associated with immune-cell patterns; no independent gland morphology.	(CO) Convergent ML selection; experimental validation was performed in blood. No independent salivary-gland clinical-cohort validation was reported.
Dai et al., 2025 [35]	Pyroptosis-related glandular signature including *PECAM1*, *IFI16*, *AIM2*, and *MPEG1*.	Lymphocytic infiltration and acinar destruction with increased candidate proteins in glandular tissue.	(IE) External public-dataset validation plus mouse and human salivary-gland experimental validation.
Chao et al., 2026 [38]	*ISG15*/*OAS1*/*LAP3*/*PARP9* signature with inflammatory macrophage state.	*ISG15* enrichment along a monocyte-to-macrophage trajectory in labial-gland single-cell data.	(IE) External public-dataset validation, glandular single-cell analysis, and functional macrophage perturbation.
Hou et al., 2026 [39]	Mitophagy phenotype with reduced *PINK1*, *GABARAPL1*, and *SQSTM1*.	Glandular expression correlated with immune-cell composition; no new morphology.	(DT) External public-dataset validation plus direct salivary-gland qRT-PCR confirmation in a small tissue series (6 SjD/6 controls); no independent clinical-cohort or functional validation.
Yin et al., 2026 [42]	Consensus ML phenotype centered on *EPSTI1*, *IFI44*, and *IFIT1*.	Human salivary-gland H&E/IHC showed inflammatory/acinar alterations and marker expression.	(IE) Internal model consensus plus independent clinical-cohort validation in a human tissue cohort.
**Pathology-based molecular subtyping**			
Hall et al., 2015 [9]	IFN-low/high states with type I-predominant, type II-predominant, and mixed IFN-high patterns.	Higher interferon activity associated with higher focus score; tissue localization supported by IHC.	(DT) No separate external validation. Direct human labial-gland molecular profiling with histopathological and IHC correlation.
Min et al., 2019 [26]	Two pathway/transcriptomic clusters with higher- versus lower-grade inflammatory profiles.	Higher-inflammatory cluster associated with more advanced glandular pathology.	(CO) Cross-public-dataset integration with pathology-score linkage and paired treatment-response subset. No independent clinical-cohort validation of the subtype was reported.
Chi et al., 2023 [10]	Clinically severe versus mild DNA-methylation subgroups.	Labial-gland epigenetic states associated with disease features; no independent morphology arm.	(DT) Internal validation within the SICCA cohort; direct labial-gland methylation with unsupervised VAE/clustering. No independent clinical-cohort validation was reported.
Cai et al., 2023 [31]	Two molecular patterns based on a ten-gene multi-tissue signature.	Tissue analyses showed different interferon/immune states; IHC localized selected proteins to inflammatory regions.	(IE) External public-dataset replication plus independent clinical-cohort validation by direct human labial-gland IHC.
Solomon et al., 2026 [40]	Multiple VAE-derived labial-gland methylation subgroups with distinct epithelial/B-cell profiles.	Subgroups differed in focus score, immune/epithelial composition, and germinal-center frequency.	(DT) Internal validation within the SICCA cohort using VAE validation and consensus clustering; no independent clinical-cohort validation.
Wu et al., 2026 [41]	Two SjD molecular subtypes derived from a shared SjD–MALT–thyroid signature.	Subtypes differed in immune-infiltration profiles; no independent histological validation.	(CO) Cross-public-dataset/cross-disease transcriptomic convergence; no independent SjD clinical-cohort validation.
**Mechanistic and translational glandular phenotypes**			
Wu et al., 2023 [29]	*IFIH1*-prioritized innate-immune phenotype.	Increased *IFIH1* in NOD submaxillary gland with lymphocytic infiltration.	(IE) Systemic human confirmation plus in vivo glandular pathological anchoring in the NOD model: IFIH1 was measured directly in submaxillary-gland tissue and accompanied by H&E evidence of lymphocytic glandular infiltration; no independent human glandular validation or functional perturbation.
Zhou et al., 2023 [30]	Hub-gene phenotype including *EZH2*, *ELAVL1*, and *IGF1R*.	EZH2 increased in glandular lymphocytes with histological grade; IGF1R acinar expression decreased with grade.	(IE) Independent clinical-cohort validation in a human labial salivary-gland H&E/IHC cohort.
Wang et al., 2025 [37]	Anoikis-related phenotype centered on *MAPK3*, *IL15*, *S100A9*, *IFI27*, and *CXCL10*.	Mouse glandular inflammation/fibrosis and epithelial apoptosis linked to the selected pathway.	(IE) External public-dataset validation plus mouse and salivary epithelial-cell validation with MAPK3 pharmacological perturbation.
Cai et al., 2026 [13]	miR-23b-3p regulatory phenotype targeting *CHUK*/IKKα-NF-κB.	Treatment reduced glandular inflammatory injury and improved AQP5/GPER and salivary flow.	(IE) Luciferase/RIP, cell-rescue experiments, exosomal delivery, and controlled mouse intervention.
Cui et al., 2026 [14]	Sphinganine-centered gland/lung lipid phenotype.	Sa exposure worsened glandular dysfunction and injury; pharmacological Myh9 inhibition produced functional rescue.	(IE) Controlled animal and cell perturbation with rescue experiments.
Mariette et al., 2022 [27]	Computationally quantified treatment-associated B-cell/plasma-cell tissue response.	Sequential belimumab plus rituximab produced the greatest CD20+ B-cell depletion and reduction in aggregate/glandular area.	(IE) Prospective multicenter clinical treatment-response assessment (not external validation of an AI-derived predictive model): randomized phase II trial with serial minor-salivary-gland biopsies and ML-assisted tissue quantification.

AI, artificial intelligence; anti-SSA, anti-Sjögren’s syndrome-related antigen A; DNA, deoxyribonucleic acid; FFPE, formalin-fixed paraffin-embedded; H&E, hematoxylin and eosin; IFN, interferon; IHC, immunohistochemistry; LSG, labial salivary gland; MALT, mucosa-associated lymphoid tissue; ML, machine learning; NF-κB, nuclear factor kappa B; NOD, non-obese diabetic; qRT-PCR, quantitative reverse-transcription polymerase chain reaction; RIP, RNA immunoprecipitation; RNA, ribonucleic acid; SICCA, Sjögren’s International Collaborative Clinical Alliance; SjD, Sjögren’s disease; TDP-43, TAR DNA-binding protein 43; VAE, variational autoencoder; WSI, whole-slide image. Validation-context indicator: IE, independent/experimental support based on validation in a separately recruited human clinical cohort and/or direct functional, experimental, or interventional validation; DT, direct or orthogonal glandular-tissue support without independent clinical-cohort or functional validation, including small confirmatory tissue series; CO, predominantly computational convergence from secondary/public datasets without independent glandular biological confirmation. External public-dataset validation alone did not qualify as IE. The indicator is descriptive and does not represent a numerical quality or certainty score. Analytical-validation setting: internal validation refers to resampling, cross-validation, or data splitting within the source cohort or dataset; external public-dataset validation refers to evaluation in a separately accessioned public dataset not used for model fitting; independent clinical-cohort validation refers to evaluation in separately recruited patients not used for model development; and prospective multicenter validation requires prospectively recruited independent participants across multiple clinical centers under a prespecified validation framework. Public datasets with known or plausible participant overlap with development data were classified as dataset reuse rather than independent external validation. These analytical-validation categories are distinct from the IE/DT/CO validation-context indicator and from biological or translational validation depth.

## Data Availability

No new data were created or analyzed in this study. Data sharing is not applicable to this article.

## Supplementary Materials

The following supporting information can be downloaded at: https://www.mdpi.com/article/10.3390/medsci14060677/s1, Table S1. Search strategies and supplementary search methods. Table S2. Full-text reports excluded and reasons for exclusion. Table S3. Risk-of-bias and methodological appraisal of included studies. Table S4. AI/ML methodological safeguards across included studies; PRISMA 2020.
