# Supplementary material for: AI-Guided Histopathological and Molecular Phenotyping of Salivary Glands in Sjögren’s Disease: Subtyping, Mechanistic Insights, and Translational Implications—A Systematic Review and Structured Functional Synthesis
Source: Med Sci (Basel). 2026 Oct 15;14(6):677. doi: 10.3390/medsci14060677 (PMC13707011; doi:10.3390/medsci14060677)
Supplement: Supplementary file 1 [file medsci-14-00677-s001.zip › Supplementary Table S1 .pdf]

Supplementary Table S1. Search strategies and supplementary search methods

Searches were conducted from database inception to 15 August 2026 without date, language, human-only, or publication-type restrictions. Database-specific syntax was used. Google Scholar was searched as a supplementary source; results were ranked by relevance and the first 200 records were screened.

<table>
<tr><th>Source</th><th>Search date</th><th>Records retrieved/screened</th><th>Limits and handling</th></tr>
<tr><td>PubMed/MEDLINE</td><td>15 Aug 2026</td><td>227</td><td>No date, language, human-only, or publication-type filters.</td></tr>
<tr><td colspan="4">Search strategy / method: ("Sjogren's Syndrome"[Mesh] OR Sjogren*[tiab] OR Sjögren*[tiab])
AND
("Artificial Intelligence"[Mesh] OR "Machine Learning"[Mesh] OR "Deep Learning"[Mesh] OR "Pattern Recognition, Automated"[Mesh] OR "artificial intelligence"[tiab] OR "machine learning"[tiab] OR "deep learning"[tiab] OR "neural network"[tiab] OR "neural networks"[tiab] OR "random forest"[tiab] OR "random forests"[tiab] OR "support vector machine"[tiab] OR "support vector machines"[tiab] OR SVM[tiab] OR LASSO[tiab] OR XGBoost[tiab] OR "gradient boosting"[tiab] OR "naive Bayes"[tiab] OR "supervised learning"[tiab] OR "unsupervised learning"[tiab] OR "hierarchical clustering"[tiab] OR "consensus clustering"[tiab] OR "feature selection"[tiab] OR "recursive feature elimination"[tiab] OR SHAP[tiab] OR "network-based"[tiab] OR "key driver analysis"[tiab] OR "systems analysis"[tiab] OR "pattern recognition"[tiab])
AND
("salivary gland"[tiab] OR "salivary glands"[tiab] OR "minor salivary gland"[tiab] OR "minor salivary glands"[tiab] OR "labial gland"[tiab] OR "labial glands"[tiab] OR parotid*[tiab] OR submandibular*[tiab] OR histopatholog*[tiab] OR histolog*[tiab] OR biopsy[tiab] OR biopsies[tiab] OR transcriptom*[tiab] OR "gene expression"[tiab] OR "single-cell"[tiab] OR "single cell"[tiab] OR molecular[tiab] OR mechanis*[tiab] OR pathway*[tiab] OR target*[tiab] OR therap*[tiab] OR treatment*[tiab] OR drug*[tiab] OR response*[tiab])</td></tr>
<tr><td>Scopus</td><td>15 Aug 2026</td><td>308</td><td>No date, language, or document-type filters; TITLE-ABS-KEY fields.</td></tr>
<tr><td colspan="4">Search strategy / method: TITLE-ABS-KEY(
(sjogren* OR sjögren*)
AND
("artificial intelligence" OR "machine learning" OR "deep learning" OR "neural network*" OR "random forest*" OR "support vector machine*" OR SVM OR LASSO OR XGBoost OR "gradient boosting" OR "naive Bayes" OR "supervised learning" OR "unsupervised learning" OR "hierarchical clustering" OR "consensus clustering" OR "feature selection" OR "recursive feature elimination" OR SHAP OR "network-based" OR "key driver analysis" OR "systems analysis" OR "pattern recognition")
AND
("salivary gland*" OR "minor salivary gland*" OR "labial salivary gland*" OR "labial gland*" OR parotid* OR submandibular* OR histopatholog* OR histolog* OR biopsy OR biopsies OR transcriptom* OR "gene expression" OR "single-cell" OR "single cell" OR molecular OR mechanis* OR pathway* OR target* OR therap* OR treatment* OR drug* OR response*)
)</td></tr>
<tr><td>Embase.com</td><td>15 Aug 2026</td><td>556</td><td>No date, language, human-only, or publication-type filters; Emtree explosion plus title/abstract/keyword terms.</td></tr>
<tr><td colspan="4">Search strategy / method: ('sjogren syndrome'/exp OR sjogren*:ti,ab,kw OR sjögren*:ti,ab,kw)
AND
('artificial intelligence'/exp OR 'machine learning'/exp OR 'deep learning'/exp OR 'pattern recognition'/exp OR 'artificial intelligence':ti,ab,kw OR 'machine learning':ti,ab,kw OR 'deep learning':ti,ab,kw OR 'neural network':ti,ab,kw OR 'neural networks':ti,ab,kw OR 'random forest':ti,ab,kw OR 'random forests':ti,ab,kw OR 'support vector machine':ti,ab,kw OR 'support vector machines':ti,ab,kw OR SVM:ti,ab,kw OR LASSO:ti,ab,kw OR XGBoost:ti,ab,kw OR 'gradient boosting':ti,ab,kw OR 'naive Bayes':ti,ab,kw OR 'supervised learning':ti,ab,kw OR 'unsupervised learning':ti,ab,kw OR 'hierarchical clustering':ti,ab,kw OR 'consensus clustering':ti,ab,kw OR 'feature selection':ti,ab,kw OR 'recursive feature elimination':ti,ab,kw OR SHAP:ti,ab,kw OR 'network-based':ti,ab,kw OR 'key driver analysis':ti,ab,kw OR 'systems analysis':ti,ab,kw OR 'pattern recognition':ti,ab,kw)
AND
('salivary gland'/exp OR 'minor salivary gland'/exp OR 'parotid gland'/exp OR 'submandibular gland'/exp OR 'salivary gland':ti,ab,kw OR 'salivary glands':ti,ab,kw OR 'minor salivary gland':ti,ab,kw OR 'minor salivary glands':ti,ab,kw OR 'labial salivary gland':ti,ab,kw OR 'labial salivary glands':ti,ab,kw OR 'labial gland':ti,ab,kw OR 'labial glands':ti,ab,kw OR parotid*:ti,ab,kw OR submandibular*:ti,ab,kw OR histopatholog*:ti,ab,kw OR histolog*:ti,ab,kw OR biopsy:ti,ab,kw OR biopsies:ti,ab,kw OR transcriptom*:ti,ab,kw OR 'gene expression':ti,ab,kw OR 'single-cell':ti,ab,kw OR 'single cell':ti,ab,kw OR molecular:ti,ab,kw OR mechanis*:ti,ab,kw OR pathway*:ti,ab,kw OR target*:ti,ab,kw OR therap*:ti,ab,kw OR treatment*:ti,ab,kw OR drug*:ti,ab,kw OR response*:ti,ab,kw)</td></tr>
<tr><td>Google Scholar</td><td>15 Aug 2026</td><td>First 200 screened; 0 additional eligible reports</td><td>No date or language filters. Results were ranked by relevance and the first 200 records were screened. No additional eligible reports were identified.</td></tr>
<tr><td colspan="4">Search strategy / method: (Sjogren OR Sjögren) salivary gland ("artificial intelligence" OR "machine learning" OR "deep learning" OR "random forest" OR LASSO OR SVM OR XGBoost OR autoencoder OR "hierarchical clustering")</td></tr>
<tr><td>Backward citation searching</td><td>Aug 2026</td><td>N/A</td><td>Reference lists of eligible and potentially eligible full-text reports were manually screened.</td></tr>
<tr><td colspan="4">Search strategy / method: No Boolean search string is applicable. Unique additional reports should be counted separately in the PRISMA “other methods” arm after final eligibility assessment.</td></tr>
<tr><td>Forward citation searching</td><td>Aug 2026</td><td>N/A</td><td>Citing records of eligible and potentially eligible reports were screened.</td></tr>
<tr><td colspan="4">Search strategy / method: No conventional Boolean search string is applicable. Unique additional reports should be counted separately in the PRISMA “other methods” arm after final eligibility assessment.</td></tr>
</table>

Abbreviations: AI, artificial intelligence; LASSO, least absolute shrinkage and selection operator; ML, machine learning; SHAP, SHapley Additive exPlanations; SVM, support vector machine.

Google Scholar note: A search string is reported for reproducibility, but it is shorter than the database strings because Google Scholar does not offer equivalent field-specific syntax and its hit counts are dynamic. Results were ranked by relevance, and the first 200 records were screened; no additional eligible reports were identified.
