# Supplementary material for: AI-Guided Histopathological and Molecular Phenotyping of Salivary Glands in Sjögren’s Disease: Subtyping, Mechanistic Insights, and Translational Implications—A Systematic Review and Structured Functional Synthesis
Source: Med Sci (Basel). 2026 Oct 15;14(6):677. doi: 10.3390/medsci14060677 (PMC13707011; doi:10.3390/medsci14060677)
Supplement: Supplementary file 1 [file medsci-14-00677-s001.zip › Supplementary Table S2 .pdf]

Supplementary Table S2. Full-text reports excluded and reasons for exclusion

Full-text eligibility was assessed against the prespecified requirement that AI/ML materially contribute to salivary-gland molecular or histopathological phenotyping, target/mechanism discovery, therapeutic prioritization, treatment stratification, or treatment-response pathology. Salivary-gland tissue had to be a primary analytical/experimental substrate, unless substantial direct glandular experimental validation supported an AI/ML-derived finding from another compartment.

Study	Complete reference	Reason for exclusion after full-text assessment
**Lili et al., 2026**	Lili C, Zhongfu T, Ming L, Huang C. Network toxicology integrated with machine learning and SHAP analysis identifies overlapping immune signatures between Di(2-ethylhexyl) phthalate (DEHP) and Sjögren's syndrome. BMC Pharmacol Toxicol. 2026;27(1):65. doi:10.1186/s40360-026-01119-x.	Environmental toxicology study focused on DEHP–Sjögren molecular overlap. AI/ML was used to characterize an exposure-related signature rather than salivary-gland pathology phenotyping, Sjögren-specific therapeutic target discovery, treatment stratification, or treatment-response pathology.
**Jiang et al., 2025**	Jiang X, Liang X, Hong Y, Hua C, Gao S, Zhang T. Machine learning-based identification of pyroptosis-related genes as biomarkers and targets in primary Sjögren's syndrome. Clin Exp Rheumatol. 2025;43(12):2172-2187. doi:10.55563/clinexprheumatol/zpd69e.	AI/ML discovery and validation were based on whole-blood/PBMC and peripheral-blood single-cell data, with molecular docking only for therapeutic prioritization; no direct salivary-gland analytical or experimental pathology arm was present.
**Liu et al., 2024**	Liu X, Wang H, Wang X, Jiang X, Jin Y, Han Y, Zhang Z. Identification and verification of inflammatory biomarkers for primary Sjögren's syndrome. Clin Rheumatol. 2024;43(4):1335-1352. doi:10.1007/s10067-024-06901-y.	The discovery datasets and experimental RT-qPCR validation were peripheral blood/PBMC based. No substantive salivary-gland tissue, molecular pathology, or histopathological validation arm was included.
**Wei et al., 2024**	Wei B, Wang A, Liu W, Yue Q, Fan Y, Xue B, Wang S. Identification of immunological characteristics and cuproptosis-related molecular clusters in primary Sjögren's syndrome. Int Immunopharmacol. 2024;126:111251. doi:10.1016/j.intimp.2023.111251.	Machine-learning discovery was performed in peripheral blood. The salivary-gland dataset was used only for secondary external validation of an already derived model, and experimental validation was serum based; therefore, the gland was not a primary analytical or experimental substrate.
**Li et al., 2024**	Li J, Xie K, Xu M, Wang Y, Huang Y, Tan T, Xie H. Significance of N6-methyladenosine RNA methylation regulators in diagnosis and subtype classification of primary Sjögren's syndrome. Heliyon. 2024;10(3):e24860. doi:10.1016/j.heliyon.2024.e24860.	Mixed blood, saliva, and salivary-gland datasets were merged for an m6A diagnostic/subtyping model without a gland-centred pathological analysis or direct glandular experimental validation; therapeutic implications remained speculative.
**Xie et al., 2024**	Xie H, Deng YM, Li JY, Xie KH, Tao T, Zhang JF. Predicting the risk of primary Sjögren's syndrome with key N7-methylguanosine-related genes: a novel XGBoost model. Heliyon. 2024;10(10):e31307. doi:10.1016/j.heliyon.2024.e31307.	The XGBoost model combined heterogeneous tissue sources and was primarily designed for disease-risk/diagnostic prediction. No direct salivary-gland experimental or histopathological validation and no eligible pathology-based therapeutic output were demonstrated.
**Zeng et al., 2022**	Zeng Q, Wen J, Zheng L, Zeng W, Chen S, Zhao C. Identification of immune-related diagnostic markers in primary Sjögren's syndrome based on bioinformatics analysis. Ann Transl Med. 2022;10(8):487. doi:10.21037/atm-22-1494.	LASSO feature selection was based on whole-blood data and qRT-PCR validation was also performed in blood. Saliva and salivary-gland datasets were used only for secondary ROC validation of previously selected genes.
**Cui et al., 2022**	Cui J, Li H, Wang T, Shen Q, Yang Y, Yu X, Hu H. Novel immune-related genetic expression for primary Sjögren's syndrome. Front Med (Lausanne). 2022;8:719958. doi:10.3389/fmed.2021.719958.	Random-forest/RFE and SVM feature selection were performed on whole-blood datasets, and RT-qPCR validation was performed in blood. The minor salivary-gland dataset was used only for secondary ROC validation.
**Laine et al., 2004**	Laine M, Virtanen I, Salo T, Konttinen YT. Segment-specific but pathologic laminin isoform profiles in human labial salivary glands of patients with Sjögren's syndrome. Arthritis Rheum. 2004;50(12):3968-3973. doi:10.1002/art.20730.	No substantive AI/ML method was used. The term "pattern recognition" referred to conventional descriptive microscopic assessment of immunofluorescence staining patterns rather than automated/computational pattern recognition.

Abbreviations: AI, artificial intelligence; ML, machine learning; PBMC, peripheral blood mononuclear cell; ROC, receiver operating characteristic; RFE, recursive feature elimination; SVM, support vector machine; WGCNA, weighted gene co-expression network analysis.
