# Supplementary material for: AI-Guided Histopathological and Molecular Phenotyping of Salivary Glands in Sjögren’s Disease: Subtyping, Mechanistic Insights, and Translational Implications—A Systematic Review and Structured Functional Synthesis
Source: Med Sci (Basel). 2026 Oct 15;14(6):677. doi: 10.3390/medsci14060677 (PMC13707011; doi:10.3390/medsci14060677)
Supplement: Supplementary file 1 [file medsci-14-00677-s001.zip › Supplementary Table S3.pdf]

## Supplementary Table S3. Risk-of-bias and methodological appraisal of included studies

Tools were matched to the primary eligible design. No numerical quality score or study-exclusion threshold was derived from these appraisals. JBI item responses are reported without an overall score; RoB 2 and PROBAST+AI overall judgments follow their respective frameworks; SYRCLE is reported item-by-item because no validated overall summary score was imposed.

### Table S3A. Revised JBI analytical cross-sectional appraisal (20 studies)

| Study | Q1 Inclusion criteria | Q2 Condition criteria | Q3 Input measurement | Q4 Outcome measurement | Q5 Confounders identified | Q6 Confounding strategy | Q7 Statistical analysis | Q8 Participants / setting | Key appraisal note |
|---|---|---|---|---|---|---|---|---|---|
| Hall et al., 2015 [9] | Y | Y | Y | Y | Y | Y | Y | Y | Standardized SICCA tissue phenotyping; blinded/independent pathology and multivariable analyses. |
| Leehan et al., 2018 [25] | Y | Y | Y | Y | Y | Y | Y | Y | Discovery/replication cohorts; blinded fibrosis assessment; age and other covariates explicitly evaluated. |
| Min et al., 2019 [26] | Y | Y | Y | Y | N | N | Y | U | Well-described multi-dataset preprocessing and validation; demographic/clinical confounding incompletely addressed. |
| Cerdó et al., 2024 [28] | U | U | Y | Y | N | N | U | U | Sparse reporting of source-cohort criteria and participant characteristics; no independent validation or confounder strategy. |
| Chi et al., 2023 [10] | Y | Y | Y | Y | Y | Y | Y | Y | Standardized SICCA procedures; methylation QC and adjustment for plate-, age-, smoking-, and ancestry-related factors. |
| Urbanski et al., 2023 [11] | Y | Y | Y | Y | Y | Y | Y | Y | Prospective direct-LSG study with prespecified eligibility and multiple potential confounders considered. |
| Wu et al., 2023 [29] | Y | Y | Y | Y | N | N | Y | Y | Clear public datasets and experimental verification; main ML discovery remained systemic without confounder adjustment. |
| Zhou et al., 2023 [30] | N | Y | Y | Y | N | N | U | Y | Independent LSG H&E/IHC validation, but data-driven post hoc exclusion of samples and no confounder handling. |
| Cai et al., 2023 [31] | Y | U | Y | Y | N | N | Y | U | Multi-dataset integration and direct LSG IHC; public-cohort diagnostic criteria/setting and confounding incompletely reported. |
| Liu et al., 2024 [32] | U | U | Y | Y | N | N | U | U | External glandular datasets used, but source-population detail, confounding, and some analytic safeguards were incompletely reported. |
| Ono-Minagi et al., 2024 [12] | Y | Y | Y | Y | Y | Y | U | Y | Explicit clinical criteria and restricted comparable groups; very small molecular/spatial subset and limited analytic detail. |
| Yang et al., 2024 [33] | U | U | Y | Y | N | N | U | U | Multiple SG datasets and standardized measurements; source-population detail/confounding limited and integrated-data split raises analytic concern. |
| Zhang et al., 2024 [34] | U | U | Y | Y | N | N | U | U | Glandular/salivary datasets with ML convergence; limited source-population detail and no clear confounder strategy. |

| Study | Q1 Inclusion criteria | Q2 Condition criteria | Q3 Input measurement | Q4 Outcome measurement | Q5 Confounders identified | Q6 Confounding strategy | Q7 Statistical analysis | Q8 Participants / setting | Key appraisal note |
|---|---|---|---|---|---|---|---|---|---|
| Dai et al., 2025 [35] | U | Y | Y | Y | Y | Y | Y | U | Independent dataset plus mouse/human-tissue validation and blinded experimental work; tissue-label/reporting inconsistency remains. |
| Wang et al., 2025 [37] | Y | U | Y | Y | N | N | U | U | Glandular component and mechanistic validation are substantial, but mixed tissue inputs and confounder control are limited. |
| Chao et al., 2026 [38] | U | U | Y | Y | N | N | Y | U | External bulk validation and central LSG single-cell arm; residual heterogeneity explicitly acknowledged and confounding not controlled. |
| Hou et al., 2026 [39] | U | U | Y | Y | N | N | Y | U | Independent LSG validation and own tissue qRT-PCR; clinical criteria and participant characteristics for tissue samples are sparse. |
| Solomon et al., 2026 [40] | Y | Y | Y | Y | Y | Y | Y | Y | Large standardized SICCA cohort; extensive methylation QC and adjustment for batch, age, smoking, and ancestry. |
| Wu et al., 2026 [41] | U | U | Y | Y | N | N | U | U | Cross-disease public datasets; residual tissue/platform confounding acknowledged; independent SjD replication lacking. |
| Yin et al., 2026 [42] | Y | Y | Y | Y | Y | Y | Y | Y | Own age-matched female tissue cohort with 2016 ACR/EULAR criteria and blinded pathology; ML discovery remains blood-centered. |

JBI codes: Y = Yes; N = No; U = Unclear. Q1–Q8 follow the revised JBI analytical cross-sectional tool. The table intentionally does not sum items into a numerical quality score.

## Table S3B. Cochrane RoB 2 appraisal of the randomized trial

| Study / tissue outcome | D1 Randomization | D2 Deviations | D3 Missing outcome data | D4 Outcome measurement | D5 Selection of reported result | Overall | Basis for judgment |
|---|---|---|---|---|---|---|---|
| Mariette et al., 2022 [27] | Some concerns | Low | **High** | Low | Low | **High** | Randomization and stratification were reported, but sequence/concealment details were not provided in the article. Blinding was extensive. Tissue analyses used paired biopsies from the completer population (n=60/86 randomized), creating substantial attrition risk for the tissue outcome. Histology/IHC quantification was standardized and treatment-blinded; tissue endpoints were prespecified. |

RoB 2 domains: D1, bias arising from the randomization process; D2, bias due to deviations from intended interventions; D3, bias due to missing outcome data; D4, bias in measurement of the outcome; D5, bias in selection of the reported result.

## Table S3C. PROBAST+AI appraisal of participant-level prediction-model studies

| Study / task | Assessment | Participants / data | Predictors | Outcome | Analysis | Overall | Applicability | Key reason |
|---|---|---|---|---|---|---|---|---|
| Duquesne et al., 2025 [8] Focus-score classification | Development quality | Low | Low | Unclear | Unclear | Unclear | Low | Multicentre development with patient-level internal validation; reference focus score came from a single local expert per centre and calibration was not reported. |
| Duquesne et al., 2025 [8] Focus-score classification | Evaluation RoB | Low | Low | Unclear | Unclear | Unclear | Low | Independent Birmingham validation is a major strength; single-centre expert reference standard and absence of calibration retain uncertainty. |
| Duquesne et al., 2025 [8] SjD classification | Development quality | Low | Low | **High** | Unclear | **High** | Low | SjD classification outcome incorporates the focus score derived from the same biopsy substrate, creating substantial incorporation/circularity concern. |
| Duquesne et al., 2025 [8] SjD classification | Evaluation RoB | Low | Low | **High** | Unclear | **High** | Low | External validation is independent, but incorporation of biopsy-derived focus score into the disease reference standard persists. |
| Ren et al., 2025 [36] Prevalent HR-OI stratification | Development quality | Low | Low | Unclear | **High** | **High** | Low | Consecutive untreated patients and a separate hospital cohort were used, but development n was small for a high-dimensional ensemble, the internal split procedure was incompletely reported, and calibration was absent. |
| Ren et al., 2025 [36] Prevalent HR-OI stratification | Evaluation RoB | Low | Low | Unclear | **High** | **High** | Low | Independent hospital validation (n=97) was performed, but the outcome was prevalent rather than future organ involvement, calibration was not reported, and uncertainty around outcome ascertainment/thresholding remains. |

PROBAST+AI terminology: for model development, Low/High/Unclear indicate concern about development quality; for model evaluation, they indicate risk of bias. Applicability is rated separately. HR-OI = high-risk extra-glandular organ involvement. The Ren model is interpreted here as cross-sectional stratification for prevalent HR-OI, not as longitudinal prognosis.

## Table S3D. SYRCLE risk-of-bias appraisal of controlled animal intervention studies

| Study | 1 Sequence generation | 2 Baseline | 3 Allocation concealment | 4 Random housing | 5 Caregiver / researcher blinding | 6 Random outcome assessment | 7 Assessor blinding | 8 Incomplete data | 9 Selective reporting | 10 Other bias | Key note |
|---|---|---|---|---|---|---|---|---|---|---|---|
| Cai et al., 2026 [13] | U | L | U | U | U | U | U | L | U | U | Animals were stated to be randomly assigned (n=6/group), but the randomization method, allocation concealment, housing randomization, and blinding were not reported. Baseline salivary-flow measurements were comparable; no attrition was evident. |
| Cui et al., 2026 [14] | U | U | U | U | U | U | U | L | U | U | Mice were stated to be randomly divided into control, experimental Sjögren’s syndrome, and experimental Sjögren’s syndrome + sphinganine groups, but sequence generation, concealment, random housing, and blinding were not described. No evident outcome attrition was reported. |

SYRCLE codes: L = low risk; H = high risk; U = unclear risk. No overall numerical score was assigned. Both preclinical studies were retained because their mechanistic and translational value is independent of the completeness of reporting of randomization and blinding safeguards.

Abbreviations: ACR/EULAR, American College of Rheumatology/European Alliance of Associations for Rheumatology; H&E, hematoxylin and eosin; HR-OI, high-risk extra-glandular organ involvement; IHC, immunohistochemistry; JBI, Joanna Briggs Institute; LSG, labial salivary gland; ML, machine learning; PROBAST+AI, Prediction model Risk Of Bias ASsessment Tool + AI; qRT-PCR, quantitative reverse transcription polymerase chain reaction; RoB, risk of bias; SG, salivary gland; SICCA, Sjögren’s International Collaborative Clinical Alliance; SjD, Sjögren’s disease; SYRCLE, Systematic Review Centre for Laboratory animal Experimentation.
