# Supplementary material for: AI-Guided Histopathological and Molecular Phenotyping of Salivary Glands in Sjögren’s Disease: Subtyping, Mechanistic Insights, and Translational Implications—A Systematic Review and Structured Functional Synthesis
Source: Med Sci (Basel). 2026 Oct 15;14(6):677. doi: 10.3390/medsci14060677 (PMC13707011; doi:10.3390/medsci14060677)
Supplement: Supplementary file 1 [file medsci-14-00677-s001.zip › Supplementary Table S4.pdf]

Supplementary Table S4. AI/ML methodological safeguards across included studies

This descriptive matrix complements design-specific risk-of-bias tools. It is not a validated score and was not used to rank or exclude studies. Codes: Y = clearly satisfied/reported; P = partially satisfied or incompletely reported; N = not satisfied/not reported; N/A = not applicable to the analytical purpose.

Study	Data provenance / cohort definition	Preprocessing / QC	Leakage / independence safeguards	Internal validation / robustness	Independent external validation	Calibration (when relevant)	Interpretability / explainability	Biological / pathological validation	Data/code accessibility	Main AI/ML methodological note
Hall et al., 2015 [9]	Y	Y	P	P	N	N/A	Y	Y	P	Interpretable tissue IFN clustering; no independent external subtype cohort.
Leehan et al., 2018 [25]	Y	Y	Y	Y	Y	N/A	Y	Y	P	Independent discovery/replication cohorts strengthen histopathologic phenotype evidence.
Min et al., 2019 [26]	Y	Y	P	Y	P	N	Y	Y	P	10-fold CV and multi-dataset integration; no formal external calibration and substantial dataset reuse.
Mariette et al., 2022 [27]	Y	Y	N/A	N/A	N/A	N/A	Y	Y	P	ML served tissue segmentation/quantification rather than patient prediction; trial design governs causal inference.
Cerdó et al., 2024 [28]	P	P	P	P	N	N	Y	N	P	Small public-dataset RF analysis with limited reporting and no independent tissue validation.
Chi et al., 2023 [10]	Y	Y	Y	Y	N	N/A	Y	P	P	VAE plus clustering with sensitivity analyses; external subtype replication absent.
Urbanski et al., 2023 [11]	Y	Y	Y	Y	P	N	Y	Y	P	Prospective direct-LSG metabolomics with train/validation/test partition; no independent-centre external cohort.
Wu et al., 2023 [29]	Y	Y	P	P	P	N/A	Y	Y	P	SVM-derived *IFIH1* supported by independent experimental glandular validation, but discovery was systemic.
Zhou et al., 2023 [30]	Y	Y	N	P	Y	N	Y	Y	P	Strong independent LSG pathology cohort, but post hoc sample exclusion creates selection/leakage concern.
Cai et al., 2023 [31]	Y	Y	P	Y	Y	N/A	Y	Y	P	Cross-dataset integration plus independent LSG IHC; extensive recurrent-GEO overlap.
Liu et al., 2024 [32]	P	Y	P	Y	Y	N	Y	N	P	External SG datasets used, but no independent wet-lab glandular validation and calibration absent.
Ono-Minagi et al., 2024 [12]	Y	Y	P	P	N	N/A	Y	Y	P	Direct digital/spatial pathology; supervised cell-classification training/validation details are limited.
Yang et al., 2024 [33]	P	Y	N	Y	P	N	Y	N	P	Integrated datasets were processed before random split, limiting independence; no tissue experiment.
Zhang et al., 2024 [34]	P	Y	P	P	N	N	Y	P	P	Feature-selection convergence but no independent glandular experimental validation.
Dai et al., 2025 [35]	P	Y	Y	Y	Y	N	Y	Y	P	Independent transcriptomic validation plus human/mouse tissue validation; minor reporting inconsistency on biopsy label.
Duquesne et al., 2025 [8]	Y	Y	Y	Y	Y	N	Y	Y	P	Strong multicentre external validation and explainable pattern discovery; calibration not reported and SjD outcome has incorporation concern.
Ren et al., 2025 [36]	Y	Y	P	Y	Y	N	Y	P	N	Independent hospital validation; small development cohort, unclear internal split details, and no calibration.
Wang et al., 2025 [37]	Y	Y	P	Y	Y	N	Y	Y	P	Public-dataset overlap remains, but mouse/cell experiments provide direct functional validation.
Cai et al., 2026 [13]	Y	Y	P	Y	N	N/A	Y	Y	Y	Small discovery n, but LASSO CV, direct target validation, rescue experiments and in-vivo therapy substantially deepen evidence.
Chao et al., 2026 [38]	Y	Y	P	Y	Y	N	Y	Y	P	External bulk validation plus central LSG single-cell mechanistic arm; residual cohort heterogeneity remains.

Study	Data provenance / cohort definition	Preprocessing / QC	Leakage / independence safeguards	Internal validation / robustness	Independent external validation	Calibration (when relevant)	Interpretability / explainability	Biological / pathological validation	Data/code accessibility	Main AI/ML methodological note
Cui et al., 2026 [14]	Y	Y	P	P	N	N	Y	Y	P	SHAP aids interpretation; causal perturbation/rescue strong, but ML training/validation safeguards are incompletely described.
Hou et al., 2026 [39]	Y	Y	P	Y	Y	N	Y	Y	P	10-fold LASSO CV, independent LSG dataset and own qRT-PCR; calibration and clinical cohort detail limited.
Solomon et al., 2026 [40]	Y	Y	Y	Y	N	N/A	Y	P	P	Large standardized LSG cohort with VAE validation and consensus resampling; no independent external subtype cohort.
Wu et al., 2026 [41]	Y	Y	P	P	P	P	Y	N	Y	Model was trained in thyroid cancer and transferred across diseases; independent SjD validation is lacking and residual tissue/platform confounding is acknowledged.
Yin et al., 2026 [42]	Y	Y	P	Y	Y	N	Y	Y	P	Own blinded parotid pathology validation; feature preselection before the blood train/validation split limits strict independence.

Interpretive note: independent external validation denotes a truly distinct cohort/dataset or independent experimental validation relevant to the AI-derived finding. Reuse of recurrent GEO or SICCA cohorts was not treated as independent replication. Calibration was considered applicable mainly to patient-level predictive/classification models; N/A does not imply superior quality.
